# Endophytes isolated from *Panax notoginseng* converted ginsenosides

**DOI:** 10.1111/1751-7915.13842

**Published:** 2021-06-03

**Authors:** Guangfei Wei, Zhongjian Chen, Bo Wang, Fugang Wei, Guozhuang Zhang, Yong Wang, Guangwei Zhu, Yuxin Zhou, Qinghe Zhao, Mingjun He, Linlin Dong, Shilin Chen

**Affiliations:** ^1^ Key Laboratory of Beijing for Identification and Safety Evaluation of Chinese Medicine Institute of Chinese Materia Medica China Academy of Chinese Medical Sciences No.16 Nanxiaojie, Dongzhimennei Ave Beijing 100700 China; ^2^ Institute of Sanqi Research Wenshan University Wenshan 663000 China; ^3^ Hubei Institute for Drug Control Wuhan 430012 China; ^4^ Wenshan Miaoxiang Notoginseng Technology, Co., Ltd. Wenshan 663000 China; ^5^ Hainan Branch Institute of Medicinal Plant Chinese Academy of Medical Sciences & Peking Union Medical College Wanning 571533 China

## Abstract

Endophytes may participate in the conversion of metabolites within medicinal plants, influencing the efficacy of host. However, the distribution of endophytes within medicinal plants *P*. *notoginseng* and how it contributes to the conversion of saponins are not well understood. Here, we determined the distribution of saponins and endophytes within *P*. *notoginseng* compartments and further confirm the saponin conversion by endophytes. We found metabolites showed compartment specificity within *P*. *notoginseng*. Potential saponin biomarkers, such as Rb1, Rg1, Re, Rc and Rd, were obtained. Endophytic diversity, composition and co‐occurrence networks also showed compartment specificity, and bacterial alpha diversity values were highest in root compartment, consistently decreased in the stem and leaf compartments, whereas those of fungi showed the opposite trend. Potential bacterial biomarkers, such as *Rhizobium*, *Bacillus*, *Pseudomonas*, *Enterobacter*, *Klebsiella*, *Pantoea* and fungal biomarkers *Phoma*, *Epicoccum*, *Xylariales*, were also obtained. Endophytes related to saponin contents were found by Spearman correlation analysis, and further verification experiments showed that *Enterobacter chengduensis* could convert ginsenoside Rg1 to F1 at a rate of 13.24%; *Trichoderma koningii* could convert ginsenoside Rb1 to Rd at a rate of 40.00% and to Rg3 at a rate of 32.31%; *Penicillium chermesinum* could convert ginsenoside Rb1 to Rd at a rate of 74.24%.

## Graphical abstract

Saponin and endophytes showed compartment‐specificity within *P. notoginseng*, and some potential biomarkers were obtained. Endophytes related to saponin contents were found by spearman correlation analysis *Enterobacter chengduensis* could convert ginsenoside Rg1 to F1 at a rate of 13.24%; *Trichoderma koningii* could convert ginsenoside Rb1 to Rd at a rate of 40.00% and to Rg3 at a rate of 32.31%; *Penicillium chermesinum* could convert ginsenoside Rb1 to Rd at a rate of 74.24%.

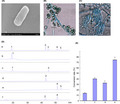

## Introduction

Endophytes localize within the inner plant parts and have profound impacts on host plants health (Larousse *et al*., 2017). The diversity and composition of endophytes are influenced by plant compartments and biogeography factors (Cregger *et al*., 2018). Each plant compartment represents a unique ecological niche for microbial entities and hosts a distinct microbial assembly as compared with other plant parts, including roots, stems, leaves, flowers and seeds (Compant *et al*., 2011). The composition of prokaryotic communities was primarily determined by the *Agave tequilana* plant compartments, whereas the composition of fungal communities was mainly influenced by the biogeography of host species (Coleman‐Derr *et al*., 2016). For *Populus*, bacterial and fungal microbiomes varied primarily across plant compartments (Beckers *et al*., 2017; Cregger *et al*., 2018). For *Cycas panzhihuaensis*, the variation of fungal community composition among different parts was obvious (Zheng *et al*., 2013). Elucidating the variations of diversity and composition in plant compartments is vital for improving crop health and productivity.

Medicinal plants are essential for improving human health. Secondary metabolites, which are the active components of herbal medicine, exert an important clinical effect (Kim *et al*., 2015). Endophytes play important roles in improving medicinal plant growth, impacting the metabolome of host plant, and thus influencing the efficacy of herbal medicine (Hassan, 2017; Huang *et al*., 2018). However, studies discussing the structural variability and niche differentiation of microbiome in medicinal plants are rare. To enhance medicinal plants growth and secondary metabolites accumulation, investigating the diversity and structure of microorganisms within the medicinal plants is essential. Some beneficial microbes could be involved in the production of bioactive metabolites; for example, artemisinin is produced by *Colletotrichum* spp., and paclitaxel is produced by *Taxomyces andreanae* (Köberl *et al*., 2013). Core microbial taxa in *Salvia miltiorrhiza* seeds promote plant growth and regulate the accumulation of the secondary metabolism (Chen *et al*., 2018). Endophytic fungi MF15, MF18, MF23 and MF24 increased the content of polysaccharide in *Anoectochilus roxburghii* by 93.5%, 100%, 89.7% and 55.1% respectively (Chen *et al*., 2005). Endophytic fungi *Aspergillus niger*, *Fusarium moniliforme* and *Trichoderma viride* isolated from *Catharanthus* *roseus* could increase the production of ajmalicine by 2–5 times (Namdeo *et al*., 2002). Thus, the rational use and manipulation of endophytes is an alternative for improving medicinal plant growth and stimulating secondary metabolites production of medicinal plants.

*Panax notoginseng* is an important medicinal plant that has therapeutic effects; it is used for public healthcare worldwide and the current market value of this species is more than $ 10 billion yearly (Sharma and Pandit, 2009). Different parts of *P*. *notoginseng* contain various types of saponins and show diverse pharmacological activities (Ng, 2006). Such as, saponins from roots, leaves and exert hemostatic, anti‐tumor, and hepatoprotective effects, respectively (Yoshikawa *et al*., 2003; Xiang *et al*., 2011). However, there are few reports on the whole saponin spectrum in different parts of *P*. *notoginseng* over a spatial scale. Endophytic bacteria *Bacillus altitudinis* and *Paenibacillus polymyxa* isolated from *P. ginseng* could improve plant growth and enhance ginsenoside accumulation (Gao *et al*., 2015; Song *et al*., 2017). Endophytes *Coniochaeta* sp. isolated from *P*. *notoginseng* can specifically convert ginsenoside Rb1 to rare saponin ginsenoside C‐K, with the conversion rate of 11.62% (Guo *et al*., 2016). These studies show that *Panax* plants endophytes are likely an important determinant of its health and secondary metabolites. Yet, there is rare information of *P*. *notoginseng* endophytic community relevant to saponins contents, especially endophytes participating in the conversion of saponin. Thus, it is valuable to study the diversity and composition of endophytes in different parts of *P*. *notoginseng* and its interactions with saponin contents. Importantly, the isolation and verification of functional microorganism converting saponin may serve as a valuable foundation for the development and application of microbial agents to improve the quality of *P*. *notoginseng*.

In this study, ultrahigh‐performance liquid chromatography mass spectrometry (UPLC‐MS) and amplicon metagenomic sequencing were carried out to determine the distribution of metabolites and endophytes in three compartments of *P*. *notoginseng* over a spatial scale respectively. Based on metabolome and metagenome data, Spearman correlation analysis was used to predict the endophytes that mainly affected the saponin contents. Traditional culture method was further used to isolate and identify culturable strains confirming their conversion function by the back grafting test. We hypothesized that (i) the distribution of endophytes and metabolites differed within plant compartments of *P*. *notoginseng*; (ii) endophytic community was relevant to saponins contents; and (iii) endophytes participated in the conversion of saponins.

## Results

### Metabolic profiling and saponin contents within plant compartments over a spatial scale

The non‐biased UPLC‐MS global metabolomics approach detected a total of 8290 peaks, of which 617 were identified as known metabolites (Dataset S1). The identified metabolites included organic acids, sugars, amino acids, polyphenols and saponins. PCA, PLS‐DA and OPLS‐DA analysis were applied to eliminate the chemical components differences in three compartments (Fig. S1A and B). A total of 609 potential biomarkers were found through one‐way ANOVA (*P* ≤ 0.05, FDR ≤ 0.05; Fig. S1D and Dataset S2). These data suggested that the chemical component distribution differed in three compartments of *P*. *notoginseng*.

**Fig. 1:**
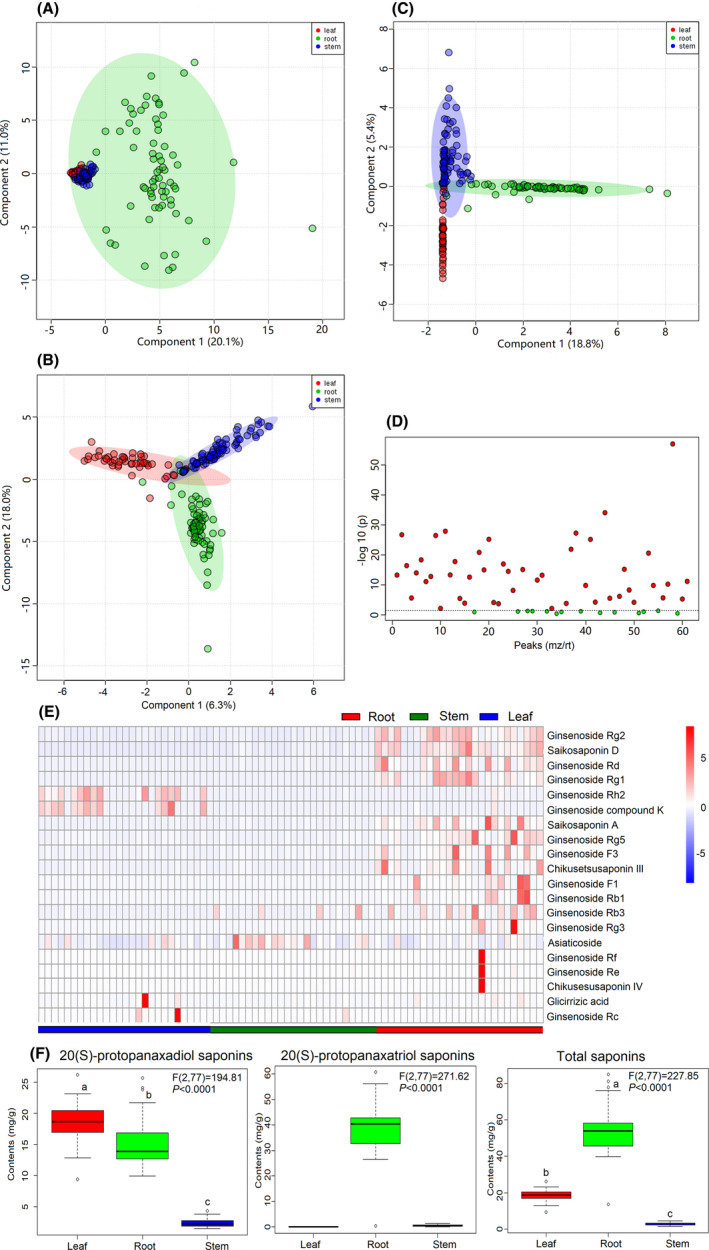
Metabolite analysis of saponins in different parts of *P*. *notoginseng*. A. PCA score plots. B. PLS‐DA score plots. C. OPLS‐DA score plots. D. One‐way ANOVA. E. Heat map of selected saponins. F. Boxplot of saponins contents.

The saponin contents and types differed in three parts of *P*. *notoginseng* (Fig. 1). PCA analysis showed that the root samples were nearly distinguished from stem and leaf samples in the principal component 1, whereas the stem and leaf data points were overlapped with each other (Fig. 1A). PLS‐DA and OPLS‐DA further highlighted the differences of saponins components in three compartments (Fig. 1B and C). A total of 61 potential saponin biomarkers were found through one‐way ANOVA (*P* ≤ 0.05, FDR ≤ 0.05; Fig. 1D and Dataset S3). Ginsenosides Rh2, compound K and Rc were abundantly present in the leaf compartments, whereas ginsenoside Rg2, Rd, Rg1, Rg5, F3, F1, Rb1, Rb3, saikosaponin D and chikusetsusaponin IV were abundantly found in the root compartments (Fig. 1E).

Based on the biomarkers within different compartments with the representative saponins (R1, Rb1, Rg1, Re, Rb2, Rc and Rd), their contents were quantified through HPLC and showed discrepancy within *P*. *notoginseng* plant compartments (Fig. 1F; Figs S2–S4). The range of PDS saponins (Rb1+Rc+Rb2+Rd) contents in the root, stem and leaf were 9.96–25.70, 1.46–4.36 and 9.41–26.18 mg g^−1^ respectively. The PTS saponin (R1+Rg1+Re) contents in the root, stem and leaf were in the ranges of 26.44–63.08 mg g^−1^, 0.24–1.32 mg g^−1^ and 0.00–0.00 mg g^−1^ respectively. The ranges of total saponins contents in the root, stem and leaf were 37.49–88.78 mg g^−1^, 1.77–5.06 mg g^−1^ and 9.41–26.18 mg g^−1^ respectively. These data suggested that PDS saponins were mainly distributed in the root and leaf compartments, while PTS saponins were chiefly located in the root compartment.

### Microbial diversity and composition within plant compartments over a spatial scale

**Fig. 2:**
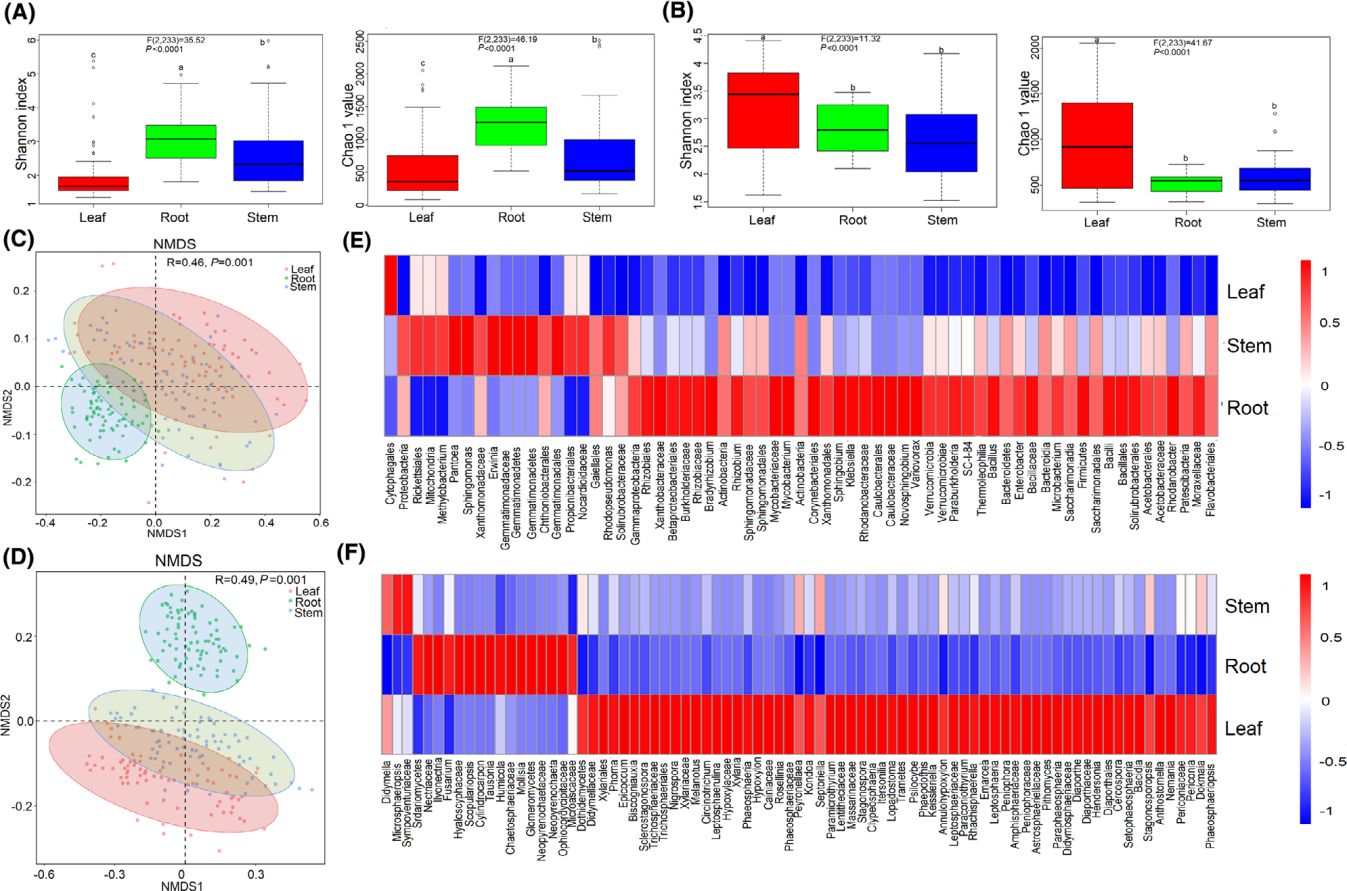
Diversity and composition of microbial communities within three compartments in *P*. *notoginseng*. A and B. Alpha diversity of bacterial and fungal communities. C and D. NMDS based on bacterial and fungal communities between plant compartments (R, ANOSIM test statistic). Data estimates represent 26 sites and are analysed by means of one‐way ANOVA comparisons. The overall plant compartment effects (F[DFn, DFd] and *P* value) are displayed at the top of each graph. E and F. Relative abundance of enriched bacterial (LDA > 2.0) and fungal (LDA > 3.0) taxa in three compartments.

Diversity and composition of bacterial microbiome in *P*. *notoginseng* showed compartment specificity at the spatial level. Approximately 6,151,304 reads were generated in 234 samples for 16S sequencing samples, and the specific number of sequences per sample was shown in Table S1. Rarefaction curves showed that the majority of root endophytic samples saturated around 400–600 OTUs, and around 150–400 OTUs, 100–200 OTUs for stem and leaf samples respectively (Fig. S5A–C). Alpha diversity values were highly dependent on plant compartments (*P* < 0.05) with high values for root samples (Chao 1, 1248.30 ± 388.85; OTU, 522.04 ± 173.73; Shannon, 3.07 ± 0.70) and consistently decreased for the stem samples (Chao 1, 737.38 ± 536.64; OTU, 317.46 ± 251.51; Shannon, 2.53 ± 0.92) and the leaf samples (Chao 1, 552.95 ± 467.18; OTU, 207.97 ± 200.96; Shannon, 1.96 ± 0.78) (Fig. 2A, Figs S6A and S7A, and Table S3). The root samples were nearly distinguished from stem and leaf samples according to NMDS and hierarchical clustering of unweighted dissimilarities analysis (*R* = 0.46, *P* = 0.001, ANOSIM test analysis) (Fig. 2C and Fig. S8A). The part‐specific OTUs were calculated to reveal the difference in the bacterial microbiome of *P*. *notoginseng* (Fig. S8B). A total of 1754, 669 and 422 OTUs were specifically exhibited in the root, stem and leaf respectively. Endophytic bacterial communities in different niches were analysed at phylum, order, family and genus level (Figs S9 and S10 and Table S4). A total of 13 phyla were detected, with Cyanobacteria and Proteobacteria being the predominant phyla accounting for more than 47.73% and 39.78% respectively. Among all bacterial phyla, Cyanobacteria, Proteobacteria, Actinobacteria, Verrucomicrobia and Planctomycetes displayed a significant plant compartments specificity (*P* < 0.05).

Alpha diversity of fungal microbiome in *P*. *notoginseng* showed compartment‐specific at the spatial level. Approximately 14 633 350 reads were generated in 234 samples for ITS sequencing samples, and the specific number of sequences per sample was shown in Table S2. Leaf samples exhibited a higher degree of variation (200–1200 OTUs) in the shape of rarefaction curves as compared to root and stem endophytic samples (400–600 OTUs) (Fig. S5D–F). Higher alpha diversity was observed in the leaf samples (Chao 1, 981.08 ± 524.81; OTU, 803.45 ± 452.44; Shannon, 3.23 ± 0.89) compared with the stem samples (Chao 1, 596.48 ± 234.76; OTU, 489.13 ± 202.93; Shannon, 2.64 ± 0.83) and the root samples (Chao 1, 436.32 ± 111.38; OTU, 436.32 ± 111.38; Shannon, 2.83 ± 0.62) (*P* < 0.05) (Fig. 2B, Figs S6B and S7B and Table S5). NMDS analyses and hierarchical clustering of unweighted dissimilarities showed that the root fungal samples were completely distinguished from stem and leaf samples (*R* = 0.49, *P* = 0.001, ANOSIM test analysis) (Fig. 2D and Fig. S11A). The part‐specific OTUs were calculated to reveal the difference in the fungal microbial of *P*. *notoginseng* (Fig. S11B). A total of 2192, 947, 3125 OTUs were specifically exhibited in the root, stem and leaf respectively. Endophytic fungal communities in different niches were analysed at phylum, order, family and genus level (Figs S12 and S13 and Table S6). Eight phyla were detected, among which Ascomycota and Basidiomycota were the predominant phyla that accounted for more than 75.94% and 6.62% respectively. All fungal phyla displayed a significant plant compartments specificity with exception of Chytridiomycota (*P* = 0.398), Cryptomycota (*P* = 0.184), Olpidiomycota (*P* = 0.020) and Zoopagomycota (*P* = 0.006).

### Microbial biomarkers obtained within plant compartments over a spatial scale

The LEfSe of bacterial OTUs and the linear discriminant analysis (LDA) demonstrated that the differences between root, stem and leaf compartments (Fig. 2E and Fig. S14). The taxa were used to generate bacterial taxonomic cladogram that illustrated the differences among plant compartments (Fig. S14A). The phyla Actinobacteria, Bacteroidetes, Verrucomicrobia, and Firmicutes, orders Flavobacteriales, Solirubrobacterales, Corynebacteriales, and Bacillales and families Mycobacteriaceae, Burkholderiaceae, Sphingomonadaceae, Rhizobiaceae and Bacillaceae were abundant in the root compartment. In the stem compartment, the significantly abundant taxa were Proteobacteria and Gemmatimonadetes at the phyla level; Gaiellales, Propionibacteriales and Gemmatimonadales at the orders level; Solirubrobacteraceae, Xanthomonadaceae and Gemmatimonadaceae at the family level. The family Cytophagales was significantly abundant in the leaf compartment. A total of 65 enriched taxa with a LDA significance threshold of 2.0 are shown in Fig. S14B. According to LDA analysis, 46, 18 and 1 bacterial groups were enriched as biomarkers in the root, stem and leaf compartments respectively (Fig. 2E).

A fungal taxonomic cladogram were generated to illustrate the differences among plant compartments (Fig. 2F and Fig. S15A). The orders Sordariomycetes, Glomeromycetes and families Neopyrenochaetaceae, Hyaloscyphaceae and Chaetosphaeriaceae were abundant in the root compartment. In the stem compartment, the significantly abundant taxa were Sympoventuriaceae at the family level. The orders Dothideomycetes, Diaporthales, Xylariales and the families Astrosphaeriellaceae, Didymellaceae, Didymosphaeriaceae were significantly abundant in the leaf compartment. The 81 enriched fungal taxa with a LDA significance threshold of 3.0 were shown in Fig. S15B. According to LDA analysis, 16, 3 and 62 fungal groups were enriched as biomarkers in the root, stem and leaf compartments respectively (Fig. 2E).

### Microbial co‐occurrence networks within plant compartments over a spatial scale

**Fig. 3:**
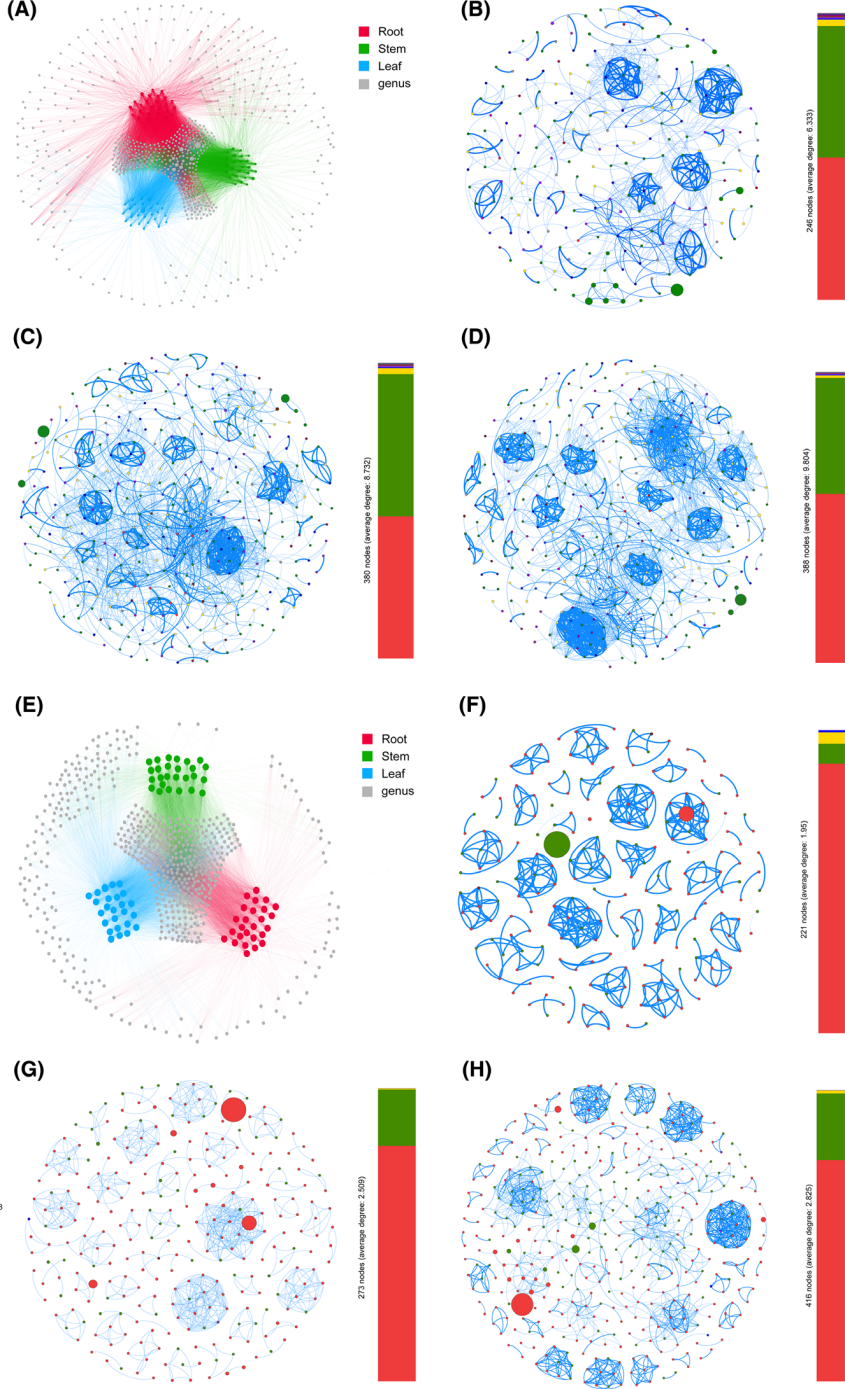
Network co‐occurrence analysis of microbial communities within plant compartments in *P*. *notoginseng*. A. Bacterial genus. B–D. Bacterial communities of root, stem and leaf respectively. E. Fungal genus. F–H. Fungal communities of root, stem and leaf respectively. A connection indicates a strong correlation (Spearman, |ρ| > 0.7, *P* < 0.01). Blue edges represent positive correlation, while red edges represent negative correlation. Each node represents taxa affiliated at genus level, and the size of node is proportional to the relative abundance of each genus. Each node is labelled at phylum level, the colour of nodes stand for different phylum.

The analysis of the co‐occurrence bacterial network showed that plant compartments had different connectivity patterns (Fig. 3A–D). The stem and leaf compartments showed a higher level of complexity and modular network than that in the root compartment. Significantly more nodes, edges, positive edges, communities and average degree were recorded in the stem (380, 1659, 1659, 30 and 8.73) and leaf (388, 1902, 1902, 31 and 9.80) compartments than that in the root compartment (246, 779, 779, 23 and 6.33) respectively (Table S7). In contrast, modularity, network diameter and average path length were significantly higher in the root compartment (0.80, 21 and 7.39) than that in the stem (0.72, 15 and 4.82) and leaf (0.75, 19 and 5.74) compartments respectively.

The co‐occurrence fungal network showed that plant compartments had different connectivity patterns (Fig. 3E–H). The root compartment showed higher level of complexity and modular network than the stem and leaf compartments. Significantly more nodes, edges, positive edges, network diameter, average path length and average degree were recorded in the leaf (416, 1175, 1175, 11, 3.99 and 8.73) and stem (273, 685, 685, 4, 1.60 and 2.51) compartments than that in the root compartment (221, 431, 431, 2, 1.02 and 1.95) respectively (Table S8). In contrast, modularity and average clustering coefficient were significantly higher in the root compartment (0.95 and 0.99) than that in the stem (0.92 and 0.93) and leaf (0.91 and 0.72) compartments respectively.

### Spearman correlation analysis of endophytes abundance and saponins contents

**Fig. 4:**
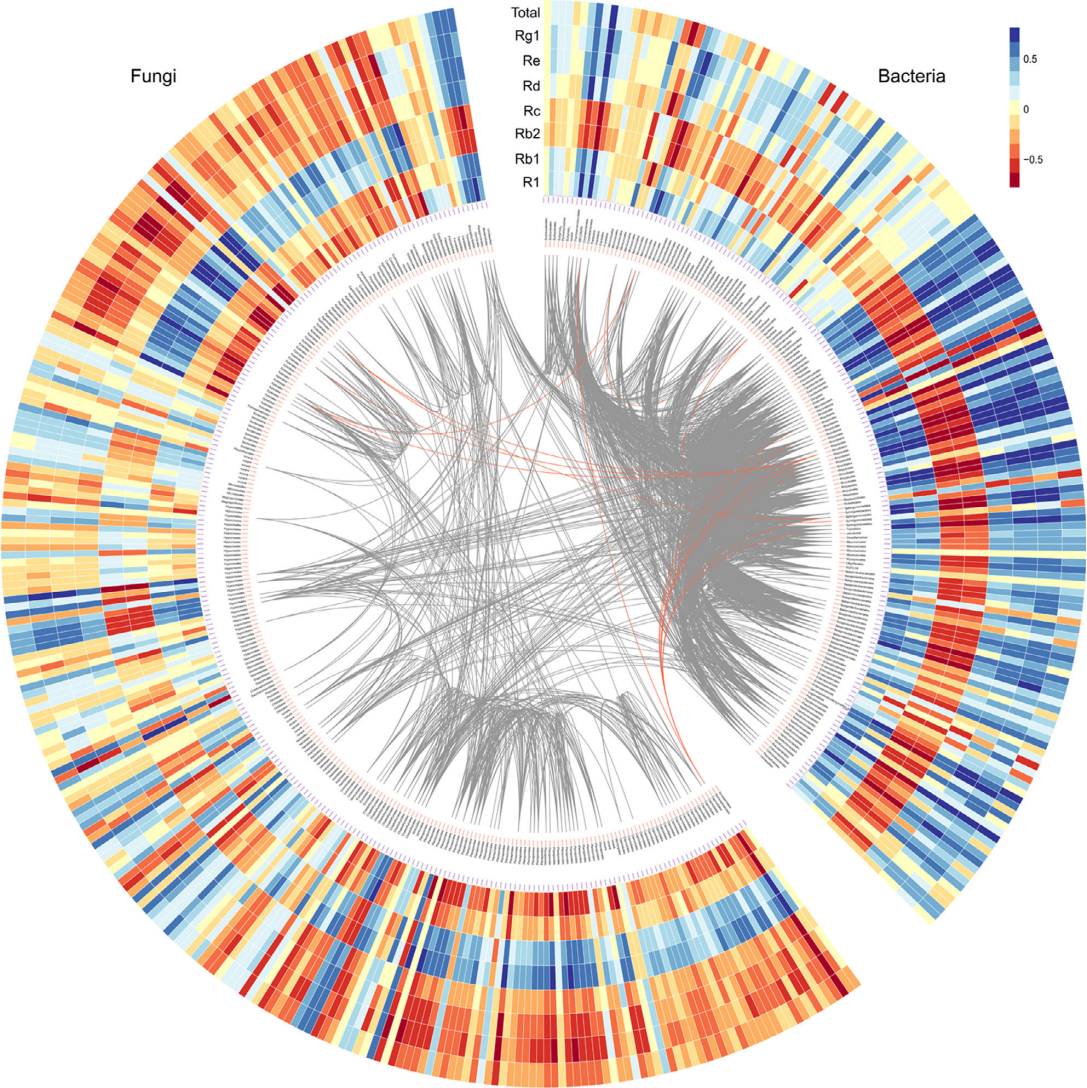
Spearman correlation of microbiome abundance and saponin contents within plant compartments in *P*. *notoginseng*. Grey and red lines indicate positive correlation between bacteria and fungi respectively.

The correlation of endophytes abundance and ginsenosides contents was estimated by Spearman rank correlation test using corr.test function in psych package, and the Spearman correlation results showed a total of 133, 140, 131, 89, 102, 221, 220 and 100 endophytes were significantly correlated with contents of R1, Rg1, Re, Rb1, Rd, Rc, Rb2 and total saponins respectively (*P* < 0.05; Fig. 4 and Dataset S4). In particular, *Enterobacter* abundance was significantly and positively correlated with R1, Rg1, Re, Rb1 and total ginsenosides contents (*P* < 0.05); *Trichoderma* and *Penicillium* abundance were significantly and positively correlated with R1, Rg1, Re, Rb1, Rd and total ginsenoside contents, whereas they were significantly and negatively correlated with Rc and Rb2 contents (*P* < 0.05). These data suggested the endophytes were related to saponin contents and might be involved in the conversion of saponins, and we further verified this function of endophytes in the following experiment.

### Endophytes within *P. notoginseng* convert saponins

**Fig. 5:**
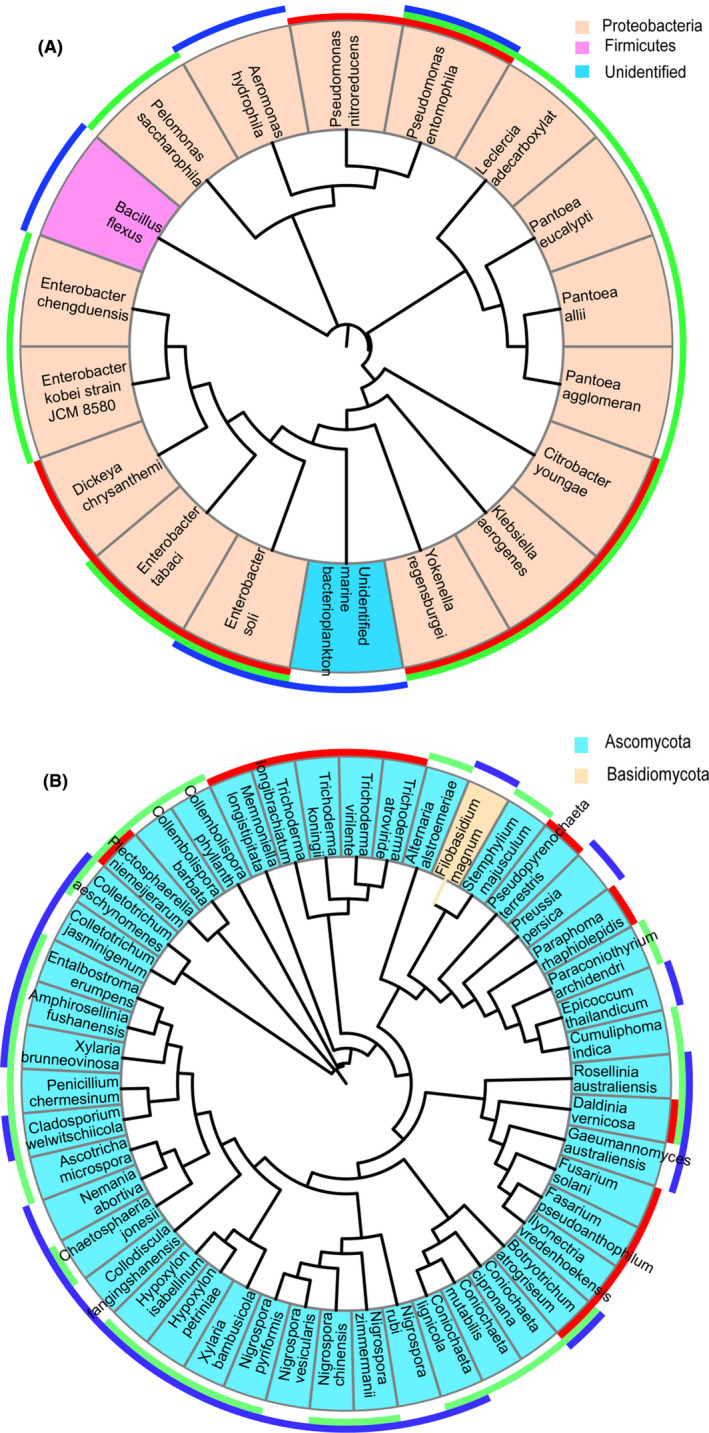
Taxonomic overlap within three compartments in culture‐dependent microbiota profiling studies. A. Bacteria. B. Fungi. Their taxonomic overlap is shown in the outer ring. The colours red, green and blue represent the root, stem and leaf respectively. Taxonomic assignment and phylogenetic tree inference were based on partial 16S and ITS2 rRNA gene sequences.

A total of 363 endophytic bacteria were isolated from *P*. *notoginseng* plants, including 120 in the root, 110 in the stem and 133 in the leaf (Dataset S5). After identification, 18 endophytic bacteria were obtained and 16 of them belonged to Proteobacteria (Fig. 5A). Among these, eight endophytic bacteria appeared in the root, while 13 in the stem, and 5 in the leaf. A total of 209 endophytic fungi (53 in the root, 79 in the stem, and 77 in the leaf) were isolated from *P*. *notoginseng* plants (Dataset S6). And 46 endophytic fungi belonging to two phyla (Ascomycota and Basidiomycota) were identified (Fig. 5B). Among, 13 endophytic fungi appeared in the root, while 27 in the stem, and 25 in the leaf.

**Fig. 6:**
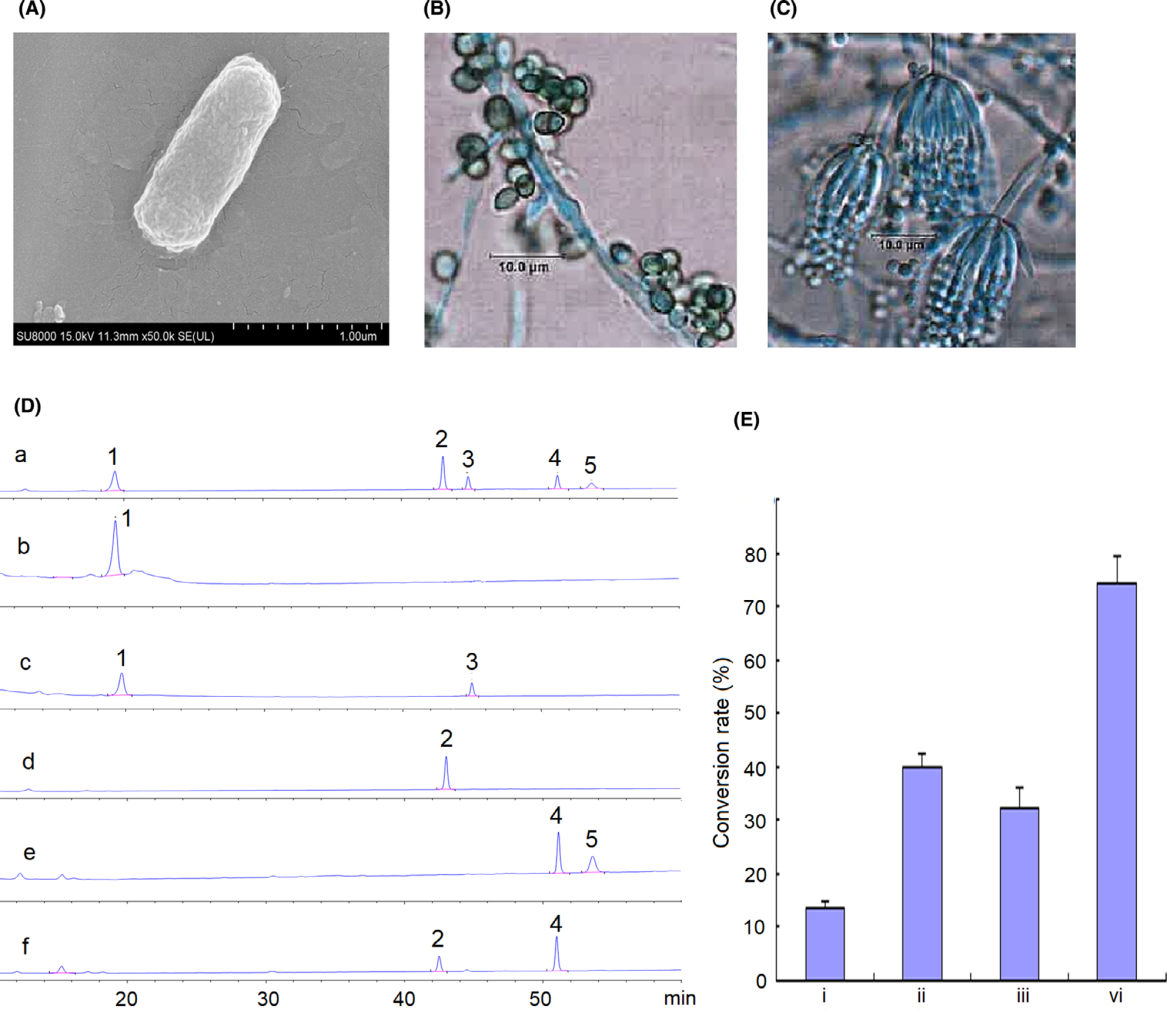
Morphology identification and conversion rate of the strain isolated from *P*. *notoginseng* plants. A. Morphology of *Enterobacter chengduensis*. B. Morphology of *Trichoderma koningii*. C. Morphology of P*enicillium chermesinum*. D. The HPLC profiles of ginsenosides. (a) Standards. 1. Rg1; 2. Rb1; 3. F1; 4. Rd; 5. Rg3. (b) Rg1 + medium control. (c) Rg1+bacterium *Enterobacter chengduensis*. (d) Rb1 + medium control. (e) Rb1+fungus *Trichoderma koningii*. (f) Rb1+fungus *Penicillium chermesinum*. E. Conversion rate (%, x ± s, *n* = 3). i. Bacterium *Enterobacter chengduensis* convert Rg1 to F1; ii. Fungus *Trichoderma koningii* convert Rg1 to Rd; iii. Fungus *Trichoderma koningii* convert Rg1 to Rg3; vi. Fungus *Penicillium chermesinum* convert Rb1 to Rd.

Then, the identified 18 endophytic bacteria and 46 endophytic fungi were cultured and infected with the sterile ginsenosides (Rg1, Re, Rc and Rb1) to verify the ginsenosides conversion ability of isolates. Following incubation, bacterium *E*. *chengduensis*, fungi *T*. *koningii* and *P*. *chermesinum* could convert saponins (Fig. 6). For bacterium *E*. *chengduensis*, is a typical rod‐shaped bacterium with a length of approximately 1–2 µm, the surface and the edge of *E*. *chengduensis* is smooth and clear without a flagellum or capsule (Fig. 6A). For fungus *T*. *koningii*, the hyphae are smooth and colourless, with a length of 2–8 µm; conidiophore are solitary, opposite or in whorls, with a length of 1–2 µm. Conidia are elliptic or subcylindrical, smooth wall, with a length of 2–4 µm (Fig. 6B). For fungus *P*. *Chermesinum*, the hyphae are smooth and colourless, with a length of 1–3 µm; conidiophore are solitary broomlike branches, with a length of 5–8 µm. Conidia are elliptic or subcylindrical, smooth wall, with a length of 1–3 µm (Fig. 6C). *E*. *chengduensis* could convert ginsenoside Rg1 to rare ginsenoside F1, and the conversion rate was 13.24% (Fig. 6D and E). *T*. *koningii* could convert ginsenoside Rb1 to ginsenoside Rd at a rate of 40.00% and rare ginsenoside Rg3 at a rate of ginsenoside 32.31%. *P*. *chermesinum* could convert ginsenoside Rb1 to ginsenoside Rd at a rate of 74.24%.

## Discussion

In our study, the distribution of metabolites showed compartment specificity, and difference of metabolic profiles within plant compartments was obtained, which was similar to the observations in the previous study (Wei *et al*., 2018a). A total of 61 potential biomarkers, including ginsenosides Rh2, Rg2, Rd, Rg1, Rg5, F3, F1, Rb1, Rb3 and Rc, were discovered in our study. Substantial studies have demonstrated anti‐oxidant (Cho *et al*., 2006), neuroprotection (Li *et al*., 2011), immunity (Huang *et al*., 2014) and anti‐inflammatory (Yu *et al*., 2017) activities of saponins. Thus, these representative saponins contents were further quantified, and the results showed that PDS saponins were mainly distributed in the root and leaf compartments, whereas PTS saponins were chiefly located in the root compartment. The varieties and contents of saponins differed among the aerial parts (flower, stem and leaf) and underground parts (root and fibril) of *P*. *notoginseng* (Wei *et al*., 2018b). *P*. *notoginseng* stems, leaves and flowers contained an abundance of 20(S)‐protopanaxadiol‐type saponins, such as notoginsenoside Fc and ginsenosides Rc, Rb2 and Rb3, which were rarely found in the roots (Wan *et al*., 2012). In contrast, the levels of ginsenosides Rb1, Rb2, Re and notoginsenoside R1 were lower in the aerial parts than that in the roots (Cui *et al*., 2009). This work provided references for rational use of different *P*. *notoginseng* parts.

Diversity of endophytes also showed compartment specificity in our *P*. *notoginseng* samples; variations of endophytic communities are also reported in previous study (Dong *et al*., 2018a). In our study, bacterial alpha diversity values were the highest in the root compartment and consistently decreased in the stem and leaf compartments, whereas fungi showed the opposite trend. The bacterial diversity of *Santiria apiculate* and *Rothmannia macrophylla* in the root samples was higher than those in the leaf samples (Haruna *et al*., 2018). In *Populus*, bacterial diversity increased from leaves, to stems and to roots, whereas fungal diversity was the greatest in stems (Cregger *et al*., 2018). From soils to epiphytes to endophytes, three crops (*Zea mays*, *Triticum aestivum* and *Hordeum vulgare*) host selection pressure sequentially increased, and bacterial diversity and network complexity consequently reduced, with the strongest effect in the leaf endosphere (Xiong *et al*., 2020). Our NMDS and hierarchical clustering analysis based on unweighted UniFrac distance showed that there were a clear boundary between the belowground compartments (root) and aboveground compartments (stem and leaf), which is consistent with other report (Beckers *et al*., 2017). All these evidences suggested that host had a strong effect on the diversity of plant‐associated microbial communities.

Cyanobacteria and Proteobacteria are the predominant bacterial phyla displayed a significant plant compartments specificity; the same phenomenon was well‐documented in *Cycas panzhihuaensis* (Zheng and Gong, 2019). Our LEfSe analyses identified some potential bacterial microbial biomarkers, such as *Rhizobium*, *Bacillus*, *Pseudomonas*, *Enterobacter*, *Klebsiella* and *Pantoea*. These biomarkers were known as plant growth‐promoting rhizobacteria, which maybe used to promote plant growth rate and enhance secondary metabolites accumulation without contaminating the environment (Vejan *et al*., 2016). Potential fungal microbial biomarkers, such as Didymella, Sordariomycetes, Phoma, Didymellaceae, were also detected in three compartments. Fungal endophytes can facilitate mineral nutrient uptake, promote plant growth and development, and induce defence resistance against pathogens (Taghinasab *et al*., 2018). In a previous *Medicago truncatula* study, LEfSe analyses identified the following: 10 biomarkers OTUs for leaves dominated by *Pseudomonas*, *Niastella* and *Phormidium*; and 12 biomarker OTUs for roots dominated by *Thioalkalibacter*, *Neorhizobium* and *Ohtaekwangia*, plus one OTU (*Ensifer*) for nodules (Brown *et al*., 2020). These results indicated that different parts of *P*. *notoginseng* could select a subset of beneficial microbes and form a unique habitat that beneficial for plant health and growth.

Network analyses have been conducted to explore microbial interaction patterns across a wide of habitats, and the differentiation in microbial co‐occurrence patterns has been well studied (Fan *et al*., 2018). Our co‐occurrence networks of endophytes within *P*. *notoginseng* showed compartment specificity, stem and leaf compartments had higher level of complexity and modular bacterial network than the root compartment, but the opposite trend was observed in fungal networks. A large complex network may facilitate more interactions and niche‐sharing (Zhou *et al*., 2020). This finding indicated that bacterial communities led to more interactions or co‐existence patterns in leaf and stem compartments, whereas the same was true for fungal communities in root compartment. Only positive links were observed in both bacterial and fungal networks within our three plant compartments. This finding highlighted the importance of microbial synergism; most microbial species cooperated with each other to resist the environmental conditions (Hoek *et al*., 2016). This indicated that plant compartments had different microbial interaction patterns and different ability to resist external environment.

Spearman correlation showed that endophytes (such as *Enterobacter*, *Trichoderma* and *Penicillium*) were significantly correlated with contents of R1, Rg1, Re, Rb1, Rd, Rc, Rb2 and total saponins in our study. Some studies have reported that endophytes as elicitors can stimulate saponins conversion. For example, endophytes *Enterobacter* sp. 20 produced ginsenosidase converting the protopanaxadiol type to ginsenoside Rg3, Rh2 and aglycone (Yu *et al*., 2008). Endophytes *Dyella* isolated from *Codonopsis pilosula* showed the strongest activities to convert major ginsenoside Rb1 to minor ginsenosides F2, C‐K and Rh1, and the maximum yield of ginsenosides F2, C‐K and Rh1 reached to 30%, 17% and 8% (Cui *et al*., 2015). Endophytes *Burkholderia* sp. GE 17‐7 isolated from *P. ginseng* can convert ginsenoside Rb1 into rare ginsenoside Rg3 (Li *et al*., 2019). Our results showed that endophytes *E*. *chengduensis* converted ginsenoside Rg1 to rare ginsenoside F1 at a rate of 13.24%, *T*. *koningii* converted ginsenoside Rb1 to Rd at a rate of 40.00% and rare ginsenoside Rg3 at a rate of 32.31%, *P*. *chermesinum* converted ginsenoside Rb1 to Rd at a rate of 74.24%, which provide new microbial resources for industrial preparation of rare ginsenoside. Previous literature reported that the hydrolytic pathways of saponins may be due to enzymes produced by microorganisms hydrolyse β‐(1‐6)‐glucoside bond on C20 or β‐(1‐2)‐glucoside bond on C3 (Cheng *et al*., 2018). 20(S)‐protopanaxadiol ginsenosides Rc, Rb1 or Rb2 can be hydrolysed to Rd, Rd is then hydrolysed to F2 or Rg3, F2 is then hydrolysed to C‐K, Rg3 can be converted to Rh2 and finally converted to diol aglycone (Noh *et al*., 2009). 20(S)‐protopanaxatriol ginsenosides Re and Rg1 can be hydrolysed to Rg2, Rh1, F1 and other secondary saponins and finally can be hydrolysed to triol aglycone (Dong *et al*., 2001). This suggested that hydrolytic pathway of ginsenoside Rg1 by our strain *E*. *chengduensis* is ginsenoside Rg1 to F1, and ginsenoside Rb1 by our strain *T*. *koningii* and *P*. *chermesinum* is Rb1 to Rd and then to Rg3. Endophytes participate in converting saponins in *Panax* plants and improving content of rare ginsenosides, which contribute to enhancing the efficacy and utilization value of *Panax* plants.

## Experimental procedures

### Plant materials

*Panax notoginseng* is mainly produced in China, Yunnan and Guangxi Provinces are the main areas for large‐scale cultivation (Meng *et al*., 2016). According to the investigation, 26 sampling sites were selected in the main production area of *P*. *notoginseng* (Dataset S1). Each sampling site had three biological replicates. Ten healthy *P*. *notoginseng* plants were collected at their harvest stage in October 2017 from each location were mixed as one biological replicate and then were separated into three compartments (leaf, stem, and root). In total, 234 samples (78 root samples, 78 stem samples and 78 leaf samples) were obtained. Each sample was mixed and divided into three subsamples, one subsample was used for metabolomic analyses, other subsample was used for metagenomic analyses, and another subsample was used for isolation of endophytes.

### Metabolites and saponins analysis

All subsamples used for metabolomic analyses were carefully washed, cut into small pieces and grounded into powder in liquid nitrogen. Metabolites were extracted with the following procedures: 0.1 g of representative samples was weighed and mixed with 1.0 ml of pure methanol (0.1% formic acid) under vortex for 10 s; the mixture was sonicated for 10 min, frozen at −20 °C for 1 h and centrifuged at 10 000 rpm for 10 min (Wei *et al*., 2018a). The upper layers were collected, filtered with 0.22 µm filter and injected into the column for metabolites using an UPLC system (Waters, UK) coupled to an electrospray ionization‐QTOF/MS apparatus (Waters, UK). The gradient was composed of water (A) and acetonitrile (B) containing 0.1% formic acid. The linear gradient was set as follows: 0–2 min, 99–80% A; 2–3 min, 80–50% A; 3–7 min, 50–20% A; 7–7.5 min, 20–1% A; 7.5–9 min, 1% A; 9–9.1 min, 1–99% A; 9.1–10 min 99% A. The column temperature was 35 °C; the flow rate was 0.4 ml min^−1^. LC‐MS raw data were transformed into MassLynx 4.1 Software (Waters, Moon Island, MA, USA) to obtain the molecular features of samples. Compounds were identified by searching against the NIST database (version). Multivariate data analysis was achieved using MetaboAnalyst 4.0 software (McGill University, Quebec, Canada) (Wei *et al*., 2020). Principal component analysis (PCA), partial least squares (PLS‐DA) and orthogonal partial least squares‐discriminant analysis (OPLS‐DA) were performed to analyse the distribution of samples. One‐way analysis of variance (ANOVA) was used to detect the difference of variance, and variance with false discovery rate (FDR) ≤ 0.05 was deemed as potential biomarkers.

Saponin quantitative analysis was performed using high‐performance liquid chromatography (HPLC, Agilent 1260 series system) (Wei *et al*., 2018a). The gradient was composed of acetonitrile (A) and water (B), and the linear gradient was set as follows: 0–12 min, 19% A; 12–60 min, 19–36% A. The column temperature was 25 °C; the flow rate was 1.0 ml min^−1^; and the wavelength was 203 nm. Statistically significant differences in saponin contents were examined using *t*‐tests in SPSS 17.0 software (SPSS Institute, Inc., Cary, NC, USA), and the boxplot was performed in R (2.15.1, The R Foundation for Statistical Computing, Vienna, Austria).

### Culture‐independent 16S and ITS2 rRNA sequencing analysis

All subsamples used for metagenomic analyses were carefully washed to clean the surface bacteria and fungi with the following procedure of immersions: 1 min in 70% (v/v) ethanol, 5 min in 5% (v/v) NaClO, 1 min in 70% (v/v) ethanol and 1 min in sterile water four times (Silvani, *et al*., 2008; Song *et al*., 2017). To check if the plants were properly surface‐sterilized, 100 µL of the last washing step water was plated on nutrient agar (NA) plate medium (containing tryptone 10.0 g, beef extract 3.0 g, NaCl 10.0 g, agar 15.0 g and pH 7.4–7.6 in 1 l of water) and potato dextrose agar (PDA) plate medium (containing potato 200 g, glucose 20 g, and agar 15 g in 1 L of water), and incubated at 37 °C and 25 °C for 7 days respectively (Carrión *et al*., 2019). Samples that properly surface‐sterilized were cut into small pieces and grounded into powder in liquid nitrogen. Total DNA was extracted from 0.1 g of samples using a FastDNA Spin Kit for Soil (MoBio Laboratories Inc., CA, USA) (Dong *et al*., 2016). The obtained 16S and ITS rRNA gene fragments were amplified using the conserved bacterial primers 27F/338R (Fierer *et al*., 2008) and fungal primers ITS1F/ITS2R (Garden and Bruns, 1993) respectively. The forward and reverse primers contained eight‐base pair barcodes respectively (Tables S1 and S2). The PCR products were purified using Agarose Gel DNA Extraction Kit (Dong *et al*., 2018b).

The DNA product was paired‐end sequenced (2 × 250 bp) with an Illumina PE 250 platform (Shanghai Biozeron Co., Ltds., China). Raw fastq files were performed using a QIIME with the following pipeline (Dong *et al*., 2018c). Raw fastq files were first demultiplexed using in‐house perl scripts according to the barcode sequences information for each sample with the following criteria: (i) the 250 bp reads were truncated at any site receiving an average quality score < 20 over a 10 bp sliding window, discarding the truncated reads that were shorter than 50 bp, (ii) exact barcode matching, 2 nucleotide mismatch in primer matching, reads containing ambiguous characters were removed, (iii) only sequences that overlap longer than 10 bp were assembled according to their overlap sequence. Reads which could not be assembled were discarded. Usearch software was used to cluster operational taxonomic units (OTU) with 97% similarity level (version 7.1 http://drive5.com/uparse/) (Edgar *et al*., 2011). Chimeric sequences were identified and removed using UCHIME. Sequences were grouped into OTUs based on 97% identity and assigned taxonomy by comparison to the SILVA (v 132) (Quast *et al*., 2013) or UNITE (v 8.2) (Kõljalg *et al*., 2013) database using the QIIME2 implementation of the Ribosomal Database Project (RDP) classifier (v 2.2) (Wang *et al*., 2007). All fastq files were submitted to National Center for Biotechnology Information (NCBI). Accession numbers were PRJNA661659 for bacteria and PRJNA661728 for fungi.

Rarefaction analysis based on mothur (v.1.21.1) was performed to identify diversity indices, including the Chao 1, OTU, and Shannon diversity indices (Schloss *et al*., 2009). Statistically significant differences in Chao 1, OTU and Shannon diversity index were examined using t‐tests in SPSS 17.0 software (SPSS Institute, Inc., 2010) and R (2.15.1, The R Foundation for Statistical Computing). One‐way ANOVA comparisons were used to test the effect of the plant compartment (root, stem and leaf) on the read abundances. Non‐metric multidimensional scaling ordination (NMDS) analysis was performed to discover the taxonomic dissimilarity between different compartments based on unweighted distance metrics, and the significance of compartments was statistically confirmed using ANOSIM (Caporaso *et al*., 2010). Linear discriminant effect size was used to characterize the features differentiating the microbial communities in plant compartments (Segata *et al*., 2011). Kruskal–Wallis sum‐rank test was performed to examine the changes and dissimilarities among classes, followed by LDA analysis to determine the size effect of each distinctively abundant taxa (Ijaz *et al*., 2018). Co‐occurrence analyses were carried out using the Python module ‘SparCC’, and networks visualization and property measurements were calculated with the interactive platform Gephi (Bastian and Jacomy). The correlation of endophytic community abundance and saponins contents was estimated by Spearman rank correlation test using corr.test function in psych package (Revelle, 2009; Sharma and Pandit, 2009).

### Isolation and identification of endophytes

Endophytes from *P*. *notoginseng* roots, stems and leaves were isolated following a modified method (Xing *et al*., 2010). Plants were thoroughly washed in running tap water for 10 min. Strips (about 5 min × 2 mm) were cut from each leaf. Stems and roots were cut into 5 mm long segments. The samples were surface‐sterilized, and sterility test was performed as previously described (Silvani, *et al*., 2008; Song *et al*., 2017; Carrión *et al*., 2019). The properly surface‐sterilized plant segments were then transferred to NA and PDA plates; PDA plates amended with 1% streptomycin to inhibit bacteria growth (Xing *et al*., 2010). NA and PDA plates were incubated at 37 °C and 25 °C for 7 days respectively.

All the bacterial and fungal colonies growing from the edges of the plant segments were transferred to new NA and PDA plates respectively. Plates were then incubated and ascertained for purity. The single bacterial and fungal colonies were numbered and used to extract rRNA gene fragments using a FastDNA Spin Kit for Soil (MoBio Laboratories Inc., USA) (Dong *et al*., 2016). The obtained 16S and ITS rRNA gene fragments were amplified using the conserved bacterial primers 27F/338R (Fierer *et al*., 2008) and fungal primers ITS1F/ITS2R (Garden and Bruns, 1993) respectively. Purification was performed and sequenced as previously described (Dong *et al*., 2018b).

The obtained sequences were compared those in GenBank (https://blast.ncbi.nlm.nih.gov/Blast.cgi), and the sequences with a similarity ≥ 99% to the partial 16S and ITS rRNA regions were considered as identical genera (Wu *et al*., 2013). Neighbour‐joining trees were constructed based on multiple sequence alignment using MEGA 5 software with 1,000 bootstrap replications (Tamura *et al*., 2011). Then, phylogenetic trees and taxonomic overlap within three compartments in culture‐dependent microbiota profiling were visualized using iTol software (Bai *et al*., 2015). Bacterial endophytes LB‐132 taken from the NA medium were fixed with glutaraldehyde 2.5% (v/v) in a 0.1 M phosphate buffer solution (PBS, pH 7.2), for 24 h at 4 °C; and then dehydrated by 10%, 30%, 50%, 70%, 85%, 95% and 100% of ethanol (Song *et al*., 2017). Scanning electron microscopy (SEM, SU8010, Hitachi, Japan) at 15.0 kV was used to observe the morphology of bacterial strains (Arroyo *et al*., 2014). Fungal endophytes SF‐85 and RF‐1 taken from the PDA medium were noted, the conidia and conidial head of fungal isolates were observed with microscope (Smart, China) by lactophenol cotton blue stain (Zheng *et al*., 2017). LB‐132, SF‐85 and RF‐1 were deposited in the China General Microbiological Culture Collection Center (CGMCC), and the collective number was CGMCC No.20650, CGMCC No.20723 and GDMCC No.20722 respectively.

### Endophytes converted ginsenosides analysis

In order to determine the effect of isolates on ginsenosides conversion, the identified isolates were cultured to infect sterile ginsenosides Rg1, Re, Rc and Rb1 (Fu *et al*., 2017). 3900 µL sterile liquid medium (NA solid medium without agar for bacterial isolates, PDA solid medium without agar for fungal isolates) with 100 µl 10 mg ml^−1^ ginsenoside were as control group. For the treatment group, 2 ml endophytes solution (OD_600_ = 0.5), 1900 µl sterile liquid medium (NA solid medium without agar for bacteria isolates, PDA solid medium without agar for fungi isolates) and 100 µl 10 mg ml^−1^ ginsenoside. After 10 days culture (160 rpm, 37 °C for bacteria and 25 °C for fungi), 600 µl disaturated n‐butanol solution was added to stop the reaction. The supernatant was concentrated in vacuum to dryness, and the residue was the dissolved in 5 ml methanol, filtered through a 0.22 µm membrane filter and analysed by HPLC (Agilent, 1260, America) (Wei *et al*., 2018a). The gradient was composed of acetonitrile (A) and water (B), and the linear gradient was set as follows: 0–12 min, 19% A; 12–60 min, 19–36% A. The column temperature was 25 °C; the flow rate was 1.0 ml min^−1^; and the wavelength was 203 nm. The peak area of each ginsenoside standard was recorded, and the standard curve was calculated according to the peak area and concentrations. The peak area of ginsenoside converted by endophytes was submitted to linear regression analysis, and the concentrations were calculated. Bioconversion rate of ginsenoside was calculated as follows (Fu *et al*., 2017): bioconversion rate of Rg1 to F1 = (Weight of Rg1 / MW of Rg1) / (Weight of F1 / MW of F1) * 100%; bioconversion rate of Rb1 to Rd = (Weight of Rb1 / MW of Rb1) / (Weight of Rd / MW of Rd) * 100%; bioconversion rate of Rb1 to Rg3 = (Weight of Rb1 / MW of Rb1) / (Weight of Rg3 / MW of Rg3) * 100%. MW is the molecular weight (Rg1 MW, 801; F1 MW, 639; Rb1 MW, 1108; Rd MW, 947; and Rg3 MW, 785).

## Conflict of interest

The authors declare no conflict of interest.

## Supporting information


**Fig. S1**. Metabolite analysis of all chemical components within different parts of *P. notoginseng*.
**Fig. S2**. Saponins contents of three parts in *P. notoginseng*.
**Fig. S3**. 20(S)‐ protopanaxadiol saponins contents within three parts in *P. notoginseng*.
**Fig. S4**. 20(S)‐ protopanaxatriol saponins contents within three parts in *P. notoginseng*.
**Fig. S5**. Rarefaction curves of detected OTUs within plant compartments in *P. notoginseng*, center values represent the median of detected OTUs.
**Fig. S6**. OTU values of microbial communities within three compartments in *P. notoginseng*.
**Fig. S7**. Diversity of microbial communities within three compartments in *P. notoginseng*.
**Fig. S8**. Hierarchical clustering and venn profiles of bacterial communities.
**Fig. S9**. The relative abundance of bacterial taxa at the phylum level within plant compartments in *P. notoginseng*.
**Fig. S10**. The relative abundance of bacterial taxa at the genus level within plant compartments in *P. notoginseng*.
**Fig. S11**. Hierarchical clustering and venn profiles of fungal communities.
**Fig. S12**. The relative abundance of fungal taxa at the phylum level within plant compartments in *P. notoginseng*.
**Fig. S13**. The relative abundance of fungal taxa at the genus level within plant compartments in *P. notoginseng*.
**Fig. S14**. Linear discriminant effect size‐identified differentially abundant bacterial taxa within plant compart.ents in *P. notoginseng*.
**Fig. S15**. Linear discriminant effect size‐identified differentially abundant fungal taxa within plant compartments in *P. notoginseng*.
**Table S1**. Bacterial barcodes and sequences numbers within plant compartments in *P. notoginseng*.
**Table S2**. Fungal barcodes and sequences numbers within plant compartments in *P. notoginseng*.
**Table S3**. Alpha diversity of bacterial communities within plant compartments in *P. notoginseng*.
**Table S4**. Phyla composition of bacterial communities within plant compartments in *P. notoginseng*.
**Table S5**. Alpha diversity of fungal communities within plant compartments in *P. notoginseng*.
**Table S6**. Phyla composition of fungal communities within plant compartments in *P. notoginseng*.
**Table S7**. Topological properties of co‐occurring bacterial networks within plant compartments calculated using the statistical Cytoscape package.
**Table S8**. Topological properties of co‐occurring fungal networks within plant compartments calculated using the statistical Cytoscape package.

**Dataset S1**. Chemical components in three parts of *Panax notoginseng*.
**Dataset S2**. Potential chemical biomarkers in three parts of *Panax notoginseng*.
**Dataset S3**. Potential saponinl biomarkers in three parts of *Panax notoginseng*.
**Dataset S4**. Spearman correlation analysis of endophytes abundance and saponins contents.
**Dataset S5**. Endophytic bacteria isolated from *P. notoginseng*.
**Dataset S6**. Endophytic fungi isolated from *P. notoginseng*.
