# Supplementary material for: Endophytes isolated from Panax notoginseng converted ginsenosides
Source: Microb Biotechnol. 2021 Jun 3;14(4):1730–46. doi: 10.1111/1751-7915.13842 (PMC8313278; doi:10.1111/1751-7915.13842)
Supplement: Supplementary file 1 — Fig. S1. Metabolite analysis of all chemical components within different parts of P. notoginseng. Fig. S2. Saponins contents of three parts in P. notoginseng. Fig. S3. 20(S)‐ protopanaxadiol saponins contents within three parts in P. notoginseng. Fig. S4. 20(S)‐ protopanaxatriol saponins contents within three parts in P. notoginseng. Fig. S5. Rarefaction curves of detected OTUs within plant compartments in P. notoginseng, center values represent the median of detected OTUs. Fig. S6. OTU values of microbial communities within three compartments in P. notoginseng. Fig. S7. Diversity of microbial communities within three compartments in P. notoginseng. Fig. S8. Hierarchical clustering and venn profiles of bacterial communities. Fig. S9. The relative abundance of bacterial taxa at the phylum level within plant compartments in P. notoginseng. Fig. S10. The relative abundance of bacterial taxa at the genus level within plant compartments in P. notoginseng. Fig. S11. Hierarchical clustering and venn profiles of fungal communities. Fig. S12. The relative abundance of fungal taxa at the phylum level within plant compartments in P. notoginseng. Fig. S13. The relative abundance of fungal taxa at the genus level within plant compartments in P. notoginseng. Fig. S14. Linear discriminant effect size‐identified differentially abundant bacterial taxa within plant compart.ents in P. notoginseng. Fig. S15. Linear discriminant effect size‐identified differentially abundant fungal taxa within plant compartments in P. notoginseng. Table S1. Bacterial barcodes and sequences numbers within plant compartments in P. notoginseng. Table S2. Fungal barcodes and sequences numbers within plant compartments in P. notoginseng. Table S3. Alpha diversity of bacterial communities within plant compartments in P. notoginseng. Table S4. Phyla composition of bacterial communities within plant compartments in P. notoginseng. Table S5. Alpha diversity of fungal communities within plant compartments in P. n [file MBT2-14-1730-s001.pdf]

Title: Endophytes isolated from *Panax notoginseng* converted ginsenosides

Running title: Endophytes converted ginsenosides

Guangfei Wei[1,★], Zhongjian Chen[2,★], Bo Wang[3,★], Fugang Wei[4], Guozhuang Zhang[1], Yong Wang[2], Guangwei Zhu[1], Yuxin Zhou[1,3], Qinghe Zhao[1], Mingjun He[5], Linlin Dong[1,*] and Shilin Chen[1,*]

[1] *Key Laboratory of Beijing for Identification and Safety Evaluation of Chinese Medicine, Institute of Chinese Materia Medica, China Academy of Chinese Medical Sciences, Beijing 100700, China*

[2] *Institute of Sanqi Research, Wenshan University, Wenshan 663000, China*

[3] *Hubei Institute for Drug Control, Wuhan 430012, China*

[4] *Wenshan Miaoxiang Notoginseng Technology, Co., Ltd., Wenshan 663000, China*

[5] *Hainan Branch Institute of Medicinal Plant, Chinese Academy of Medical Sciences & Peking Union Medical College, Wanning 571533, China*

E-mail addresses:

Guangfei Wei: gfwei@icmm.ac.cn

Zhongjian Chen: 18687656337@126.com

Bo Wang: wang_bo1986@hotmail.com

Fugang Wei: weifugang@live.com

Guozhuang Zhang: zgz123zgz@163.com

Yong Wang: ws-wangyong37@163.com

Guangwei Zhu: gwzhu@icmm.ac.cn

Yuxin Zhou: zyx123hn@163.com

Qinghe Zhao: qhzhao@icmm.ac.cn

Mingjun He: mjhe@implad.ac.cn

*** Corresponding author**

TEL: (+86) 18911917789; fax: (+86) 1062899776; email: lldong@icmm.ac.cn

*** *Corresponding author**

TEL: (+86) 1057203877; fax: (+86) 1062899776; email: slchen@icmm.ac.cn

Present address: No.16 Nanxiaojie, Dongzhimennei Ave. Beijing 100700, China.

Author contributions

★These authors contributed equally.

Supporting Information

A. Supporting Figures

Fig. S1 Metabolite analysis of all chemical components within different parts of *P. notoginseng*.

Fig. S2 Saponins contents of three parts in *P. notoginseng*.

Fig. S3 20(S)- protopanaxadiol saponins contents within three parts in *P. notoginseng*.

Fig. S4 20(S)- protopanaxatriol saponins contents within three parts in *P. notoginseng*.

Fig. S5 Rarefaction curves of detected OTUs within plant compartments in *P. notoginseng*, center values represent the median of detected OTUs.

Fig. S6 OTU values of microbial communities within three compartments in *P. notoginseng*.

Fig. S7 Diversity of microbial communities within three compartments in *P. notoginseng*.

Fig. S8 Hierarchical clustering and venn profiles of bacterial communities.

Fig. S9 The relative abundance of bacterial taxa at the phylum level within plant compartments in *P. notoginseng*.

Fig. S10 The relative abundance of bacterial taxa at the genus level within plant compartments in *P. notoginseng*.

Fig. S11 Hierarchical clustering and venn profiles of fungal communities.

Fig. S12 The relative abundance of fungal taxa at the phylum level within plant compartments in *P. notoginseng*.

Fig. S13 The relative abundance of fungal taxa at the genus level within plant compartments in *P. notoginseng*.

Fig. S14 Linear discriminant effect size-identified differentially abundant bacterial taxa within plant compartments in *P. notoginseng*.

Fig. S15 Linear discriminant effect size-identified differentially abundant fungal taxa within plant compartments in *P. notoginseng*.

B. Supporting Tables

Table S1 Bacterial barcodes and sequences numbers within plant compartments in *P. notoginseng*.

Table S2 Fungal barcodes and sequences numbers within plant compartments in *P. notoginseng*.

Table S3 Alpha diversity of bacterial communities within plant compartments in *P. notoginseng*.

Table S4 Phyla composition of bacterial communities within plant compartments in *P. notoginseng*.

Table S5 Alpha diversity of fungal communities within plant compartments in *P. notoginseng*.

Table S6 Phyla composition of fungal communities within plant compartments in *P. notoginseng*.

Table S7 Topological properties of co-occurring bacterial networks within plant compartments calculated using the statistical Cytoscape package.

Table S8 Topological properties of co-occurring fungal networks within plant compartments calculated using the statistical Cytoscape package.

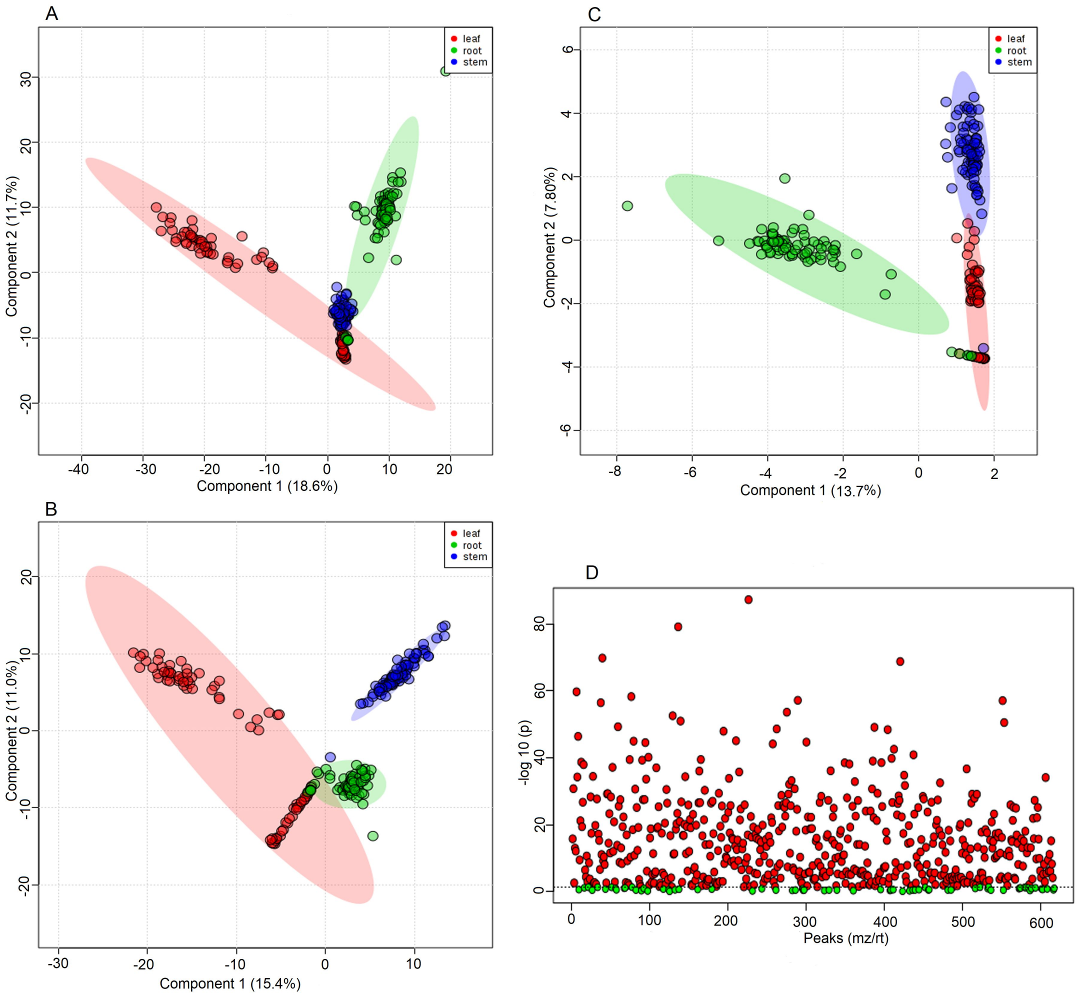

Fig. S1 Metabolite analysis of all chemical components within different parts of *P. notoginseng*. (A). PCA score plots. (B). PLS-DA score plots. (C). OPLS-DA score plots. (D). One-way ANOVA. PCA, principal Component Analysis; PLS-DA, partial least squares-discriminant analysis; OPLS-DA, orthogonal partial least squares-discriminant analysis; ANOVA, analysis of variance.

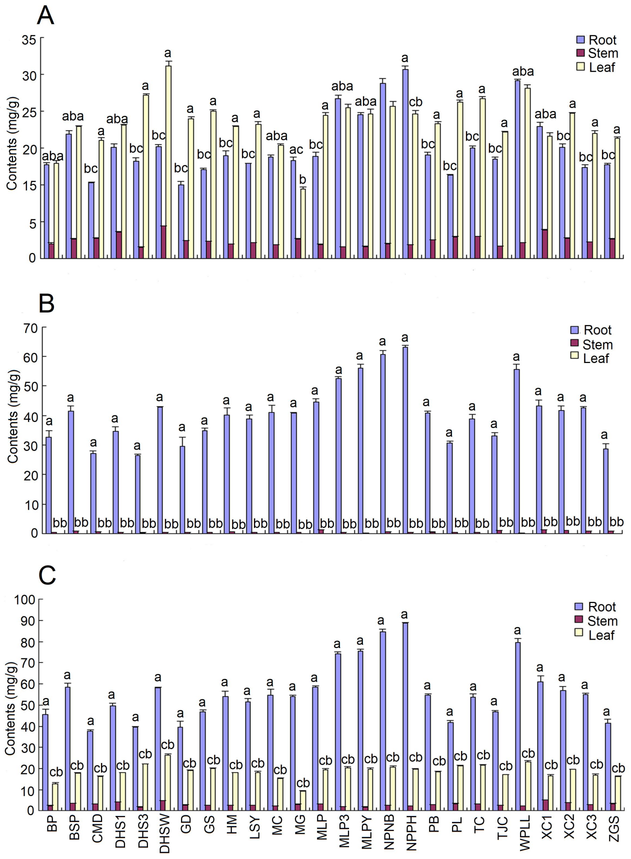

Fig. S2 Saponins contents of three parts in *P. notoginseng* over a spatial scale. (A). Total amount of 20(S)- protopanaxadiol saponins (PDS) contents, including ginsenosides Rb1, Rc, Rb2, and Rd. (B). Total amount of 20 (S)- protopanaxatriol saponins (PTS) contents, including notoginsenoside R1, ginsenoside Rg1 and Re. (C) Total saponins contents. Significance differences (P<0.05) across plant compartments were indicated with *lowercase letters*. Data represent the mean ± SD (n = 3). BP, Banpo village; BSP, Beishapo village; CMD, Chengmendong village; DHS1, Daheishan village 1; DHS3, Daheishan village 3; DHSW, Daheishan village (no pesticide); GD, Gende village; GS, Guishan village; HM, Hemo village; LSY, Laosaiyi village; MC, Meichang village; MG, Maguan village; MLP1, Masupo village 1; MLP3, Masupo village 3; MLPY, Masupo village; NPNB, Naponabing village; NPPH, Napopohe village; PB, Pingba village; PL, Panlong village; TC, Tiechang village; TJC, Tongjiachong village; WPLL, Wupingleilong village; XC1, Xichou village; XC2, Xichou village 2; XC3, Xichou village 3; ZGS, Zhiguoshan village.

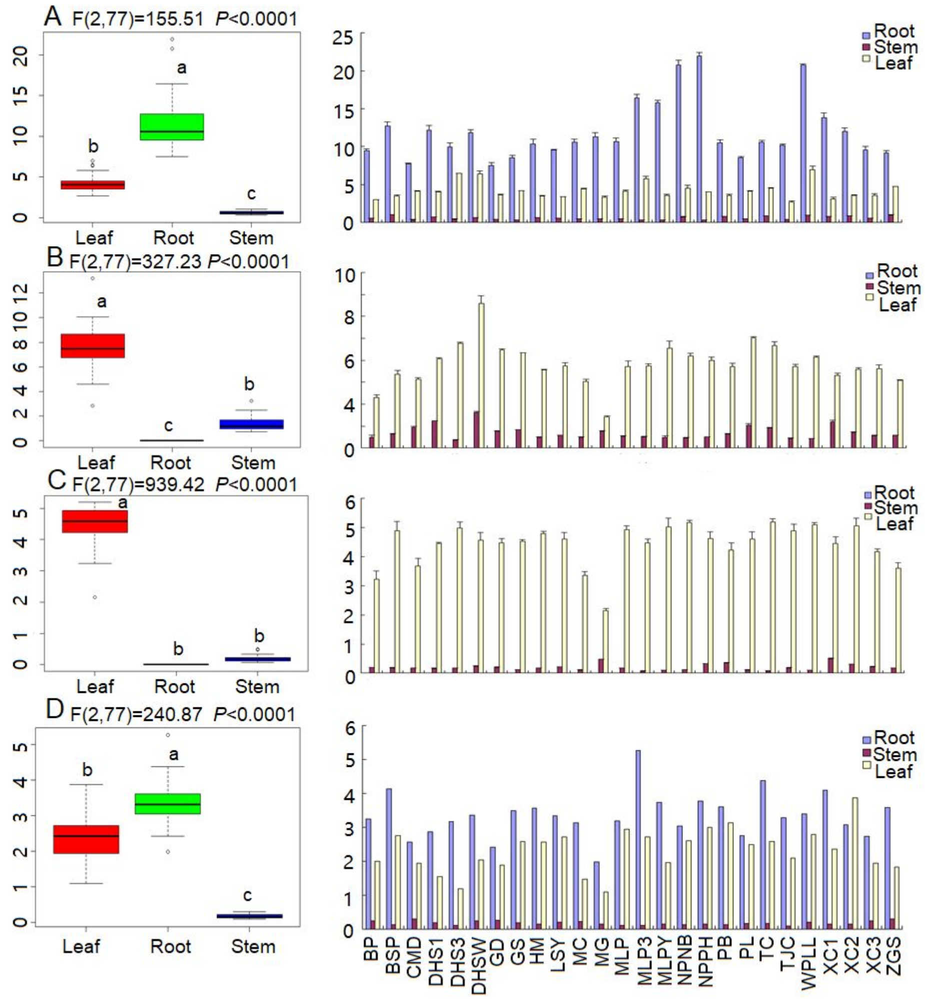

Fig. S3 20(S)- protopanaxadiol saponins contents within three parts in *P. notoginseng*. (A) Ginsenoside Rb1 contents. (B) Ginsenoside Rc contents. (C) Ginsenoside Rb2 contents. (D) Ginsenoside Rd contents. Significance differences (P<0.05) across plant compartments were indicated with *lowercase letters*. Data represent the mean ± SD (n = 3). BP, Banpo village; BSP, Beishapo village; CMD, Chengmendong village; DHS1, Daheishan village 1; DHS3, Daheishan village 3; DHSW, Daheishan village (no pesticide); GD, Gende village; GS, Guishan village; HM, Hemo village; LSY, Laosaiyi village; MC, Meichang village; MG, Maguan village; MLP1, Masupo village 1; MLP3, Masupo village 3; MLPY, Masupo village; NPNB, Naponabing village; NPPH, Napopohe village; PB, Pingba village; PL, Panlong village; TC, Tiechang village; TJC, Tongjiachong village; WPLL, Wupingleilong village; XC1, Xichou village; XC2, Xichou village 2; XC3, Xichou village 3; ZGS, Zhiguoshan village.

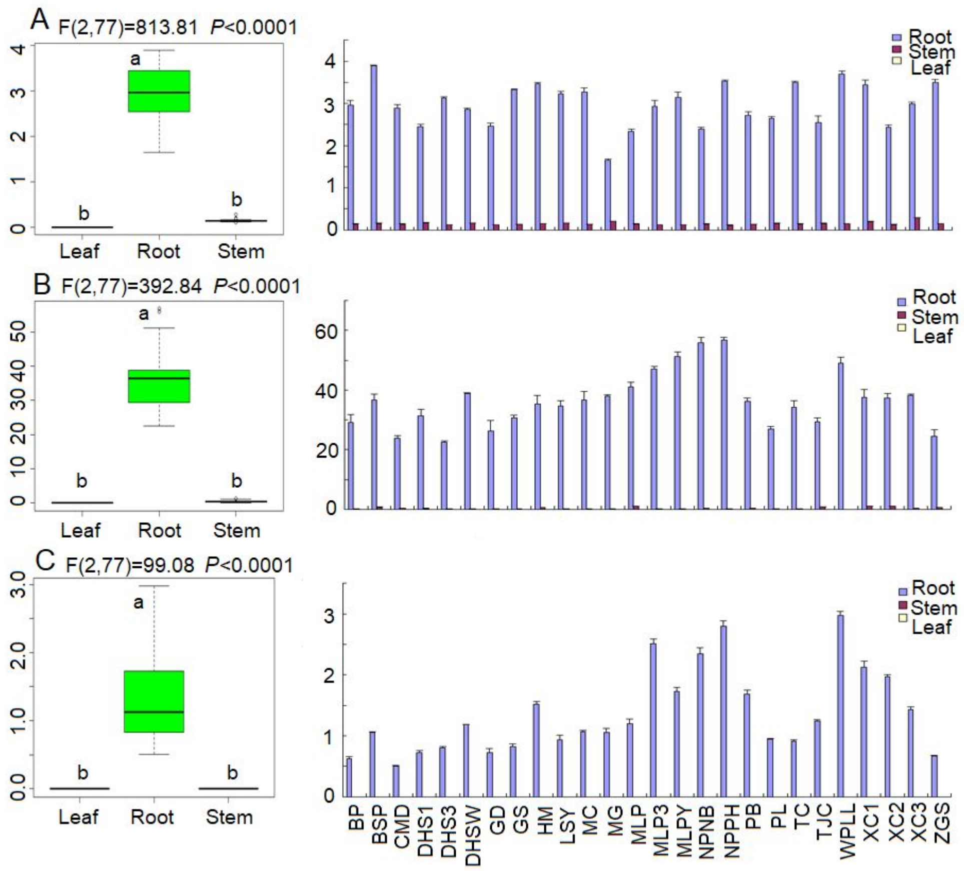

Fig. S4 20(S)- protopanaxatriol saponins contents within three parts in *P. notoginseng*. (A) Notoginsenoside R1 contents. (B) Ginsenoside Rg1 contents. (C) Ginsenoside Re contents. Significance differences (P<0.05) across plant compartments were indicated with *lowercase letters*. Data represent the mean ± SD (n = 3). BP, Banpo village; BSP, Beishapo village; CMD, Chengmendong village; DHS1, Daheishan village 1; DHS3, Daheishan village 3; DHSW, Daheishan village (no pesticide); GD, Gende village; GS, Guishan village; HM, Hemo village; LSY, Laosaiyi village; MC, Meichang village; MG, Maguan village; MLP1, Masupo village 1; MLP3, Masupo village 3; MLPY, Masupo village; NPNB, Naponabing village; NPPH, Napopohe village; PB, Pingba village; PL, Panlong village; TC, Tiechang village; TJC, Tongjiachong village; WPLL, Wupingleilong village; XC1, Xichou village; XC2, Xichou village 2; XC3, Xichou village 3; ZGS, Zhiguoshan village.

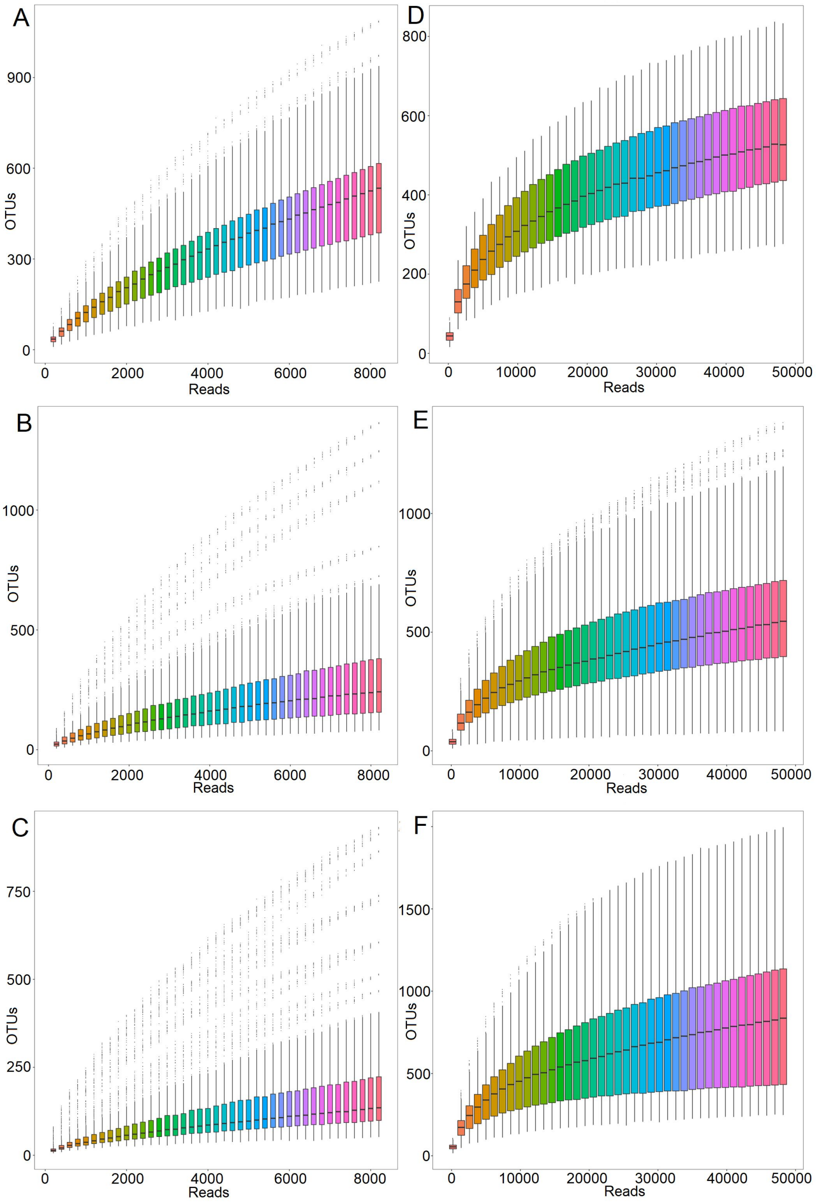

Fig. S5 Rarefaction curves of detected OTUs within plant compartments in *P. notoginseng*, center values represent the median of detected OTUs. (A). Bacterial OTUs of root. (B). Bacterial OTUs of stem. (C). Bacterial OTUs of leaf. (D). Fungal OTUs of root. (E). Fungal OTUs of stem. (F). Fungal OTUs of leaf.

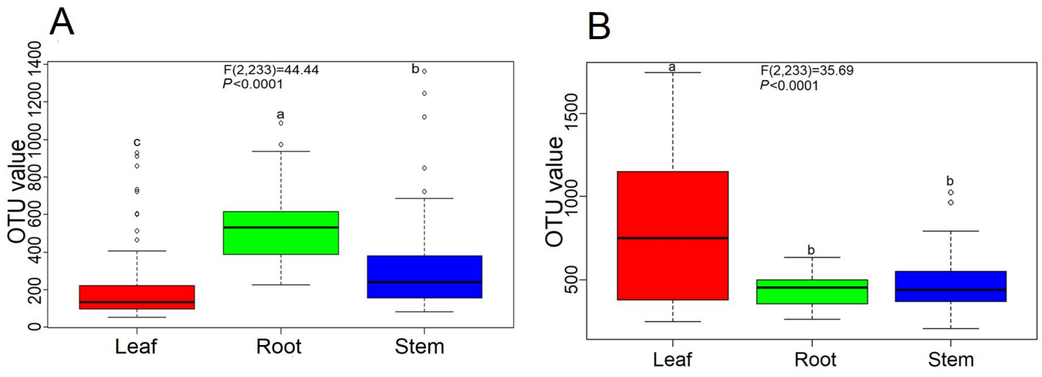

Fig. S6 OTU values of microbial communities within three compartments in *P. notoginseng*. (A). Bacteria. (B). Fungi.

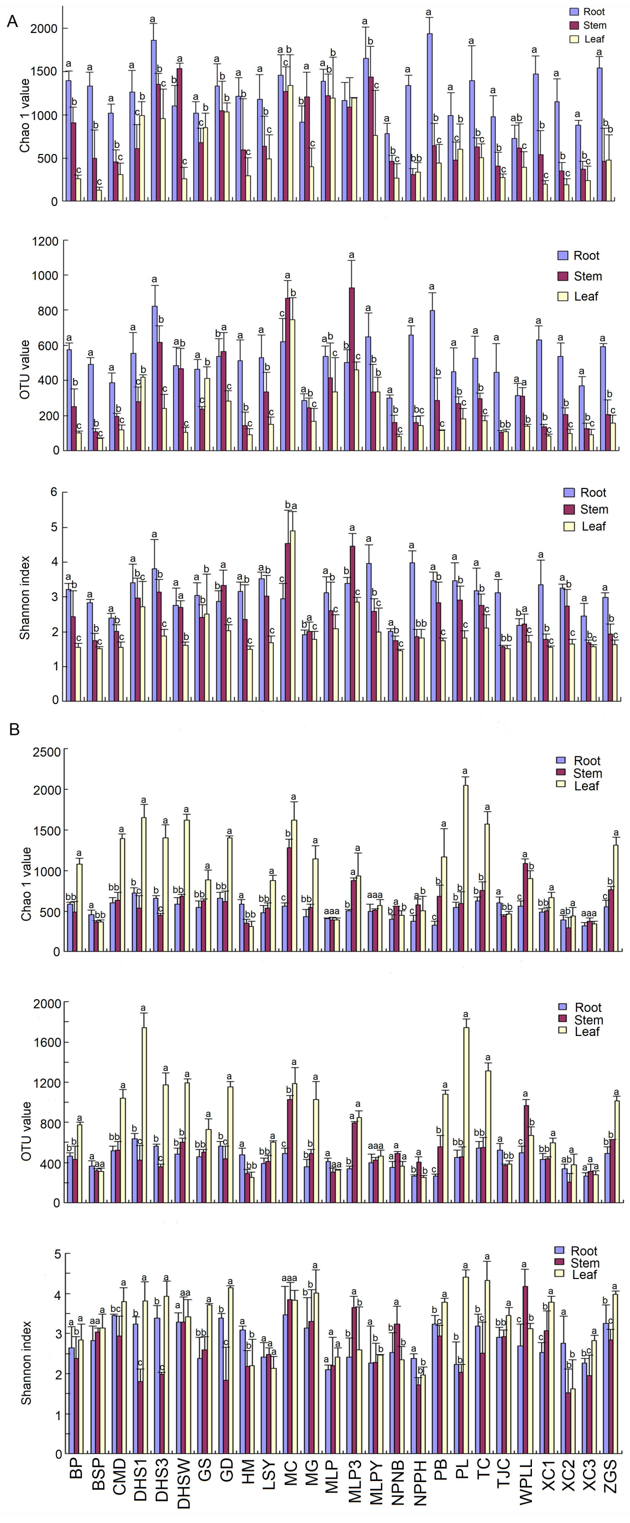

Fig. S7 Diversity of microbial communities within three compartments in *P. notoginseng* over a spatial scale. (A). Alpha diversity of bacterial communities. (B). Alpha diversity of fungal communities. Significance differences (P<0.05) across plant compartments were indicated with *lowercase letters*. Data represent the mean ± SD (*n*

= 3). BP, Banpo village; BSP, Beishapo village; CMD, Chengmendong village; DHS1, Daheishan village 1; DHS3, Daheishan village 3; DHSW, Daheishan village (no pesticide); GD, Gende village; GS, Guishan village; HM, Hemo village; LSY, Laosaiyi village; MC, Meichang village; MG, Maguan village; MLP1, Masupo village 1; MLP3, Masupo village 3; MLPY, Masupo village; NPNB, Naponabing village; NPPH, Napopohe village; PB, Pingba village; PL, Panlong village; TC, Tiechang village; TJC, Tongjiachong village; WPLL, Wupingleilong village; XC1, Xichou village; XC2, Xichou village 2; XC3, Xichou village 3; ZGS, Zhiguoshan village.

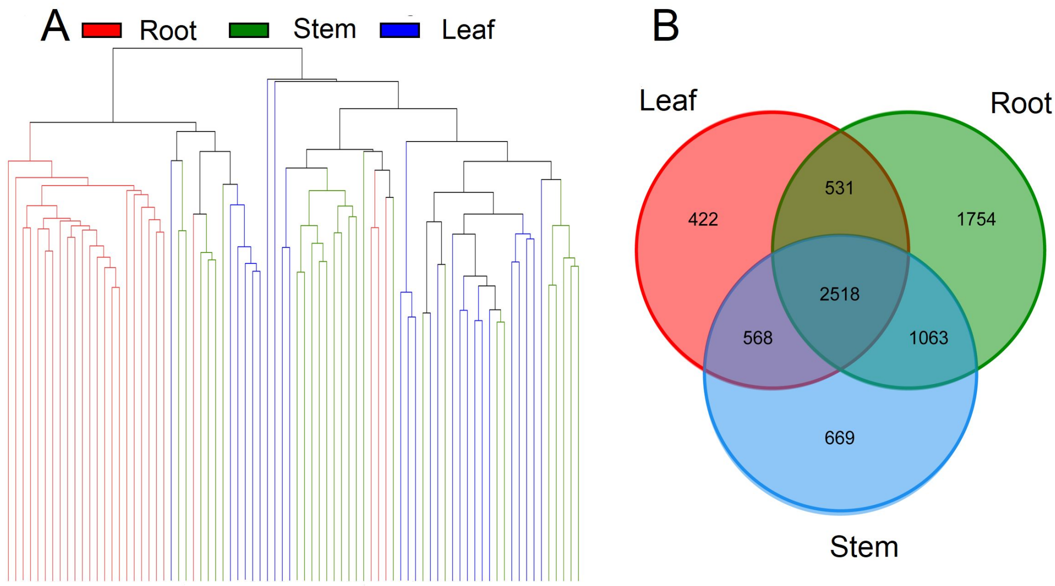

Fig. S8 Hierarchical clustering and venn profiles of bacterial communities. (A). Hierarchical clustering of the samples based on bacterial communities unweighted dissimilarity. (B) Venn profiles of bacterial communities.

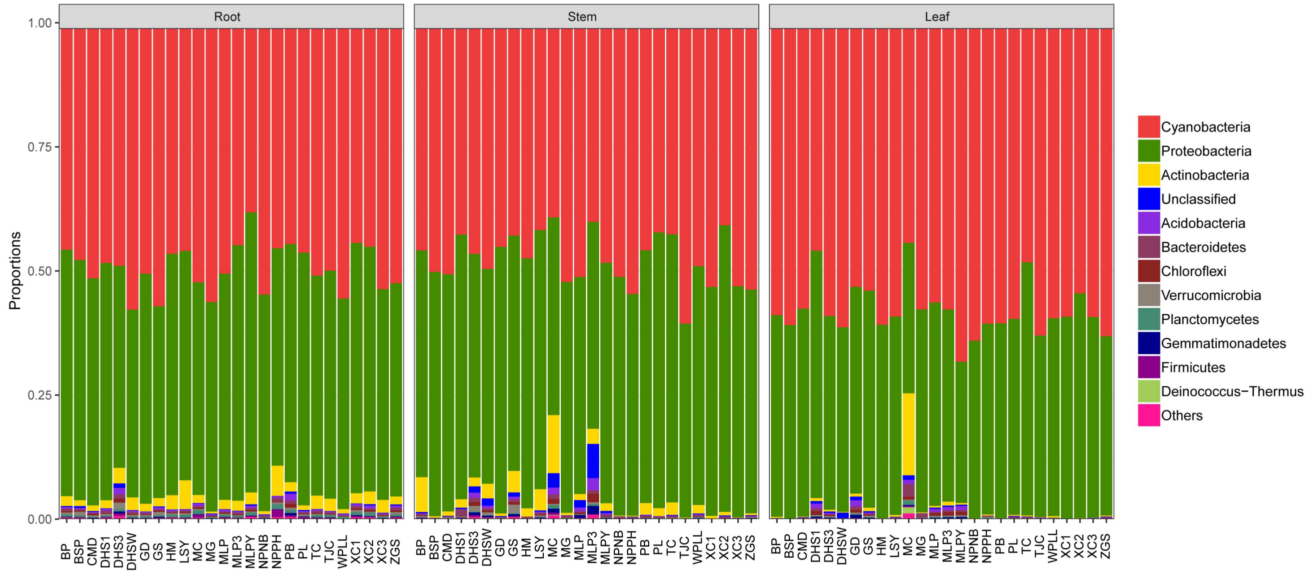

Fig. S9 The relative abundance of bacterial taxa at the phylum level within plant compartments in *P. notoginseng*. BP, Banpo village; BSP, Beishapo village; CMD, Chengmendong village; DHS1, Daheishan village 1; DHS3, Daheishan village 3; DHSW, Daheishan village (no pesticide); GD, Gende village; GS, Guishan village; HM, Hemo village; LSY, Laosaiyi village; MC, Meichang village; MG, Maguan village; MLP1, Masupo village 1; MLP3, Masupo village 3; MLPY, Masupo village; NPNB, Naponabing village; NPPH, Napopohe village; PB, Pingba village; PL, Panlong village; TC, Tiechang village; TJC, Tongjiachong village; WPLL, Wupingleilong village; XC1, Xichou village; XC2, Xichou village 2; XC3, Xichou village 3; ZGS, Zhiguoshan village.

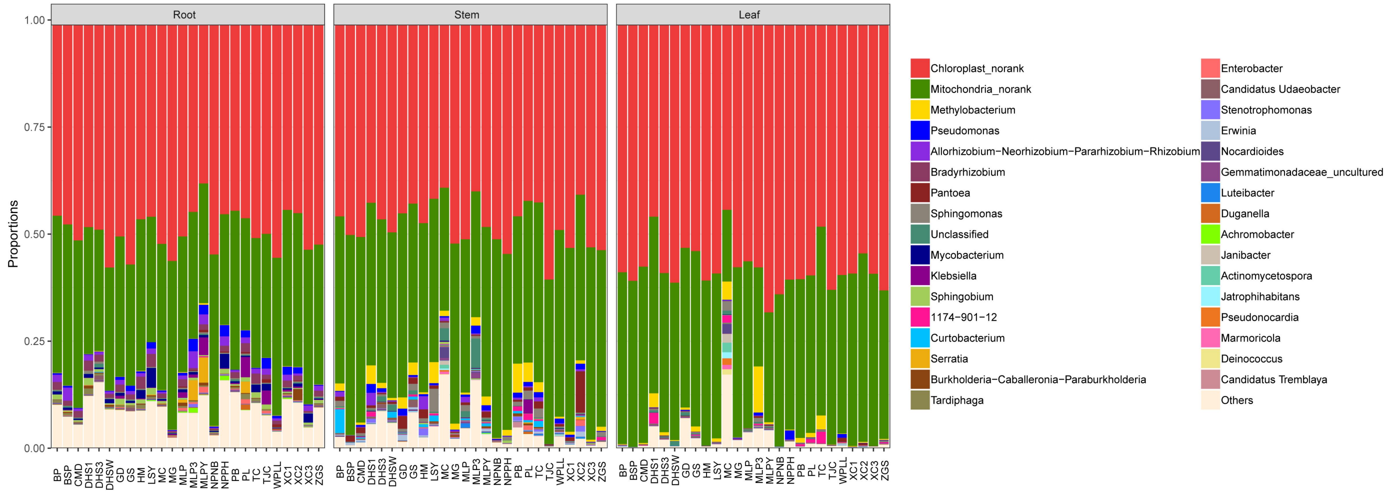

Fig. S10 The relative abundance of bacterial taxa at the genus level within plant compartments in *P. notoginseng*. BP, Banpo village; BSP, Beishapo village; CMD, Chengmendong village; DHS1, Daheishan village 1; DHS3, Daheishan village 3; DHSW, Daheishan village (no pesticide); GD, Gende village; GS, Guishan village; HM, Hemo village; LSY, Laosaiyi village; MC, Meichang village; MG, Maguan village; MLP1, Masupo village 1; MLP3, Masupo village 3; MLPY, Masupo village; NPNB, Naponabing village; NPPH, Napopohe village; PB, Pingba village; PL, Panlong village; TC, Tiechang village; TJC, Tongjiachong village; WPLL, Wupingleilong village; XC1, Xichou village; XC2, Xichou village 2; XC3, Xichou village 3; ZGS, Zhiguoshan village.

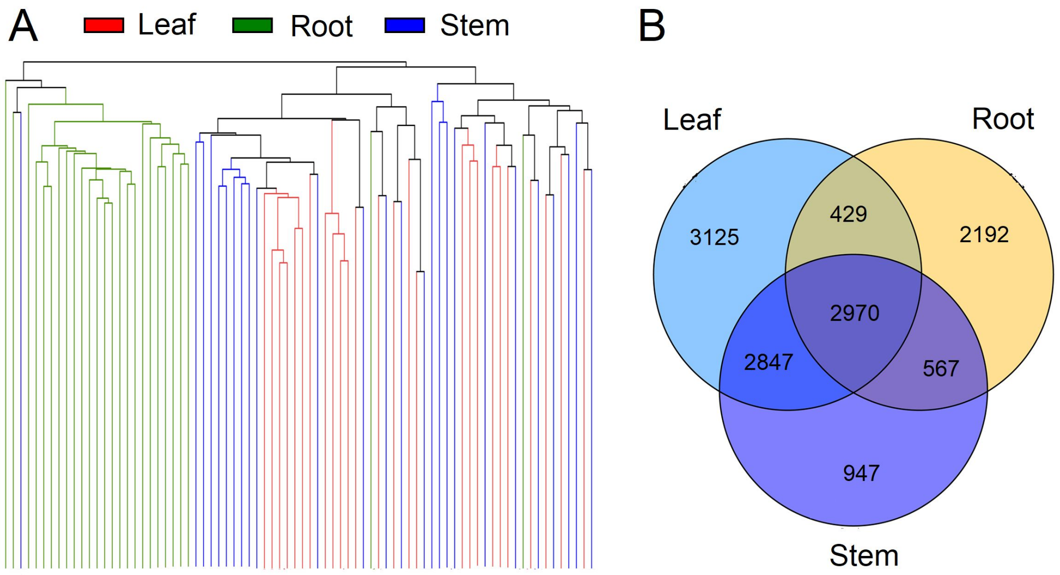

Fig. S11 Hierarchical clustering and venn profiles of fungal communities. (A). Hierarchical clustering of the samples based on fungal communities unweighted dissimilarity. (B) Venn profiles of fungal communities.

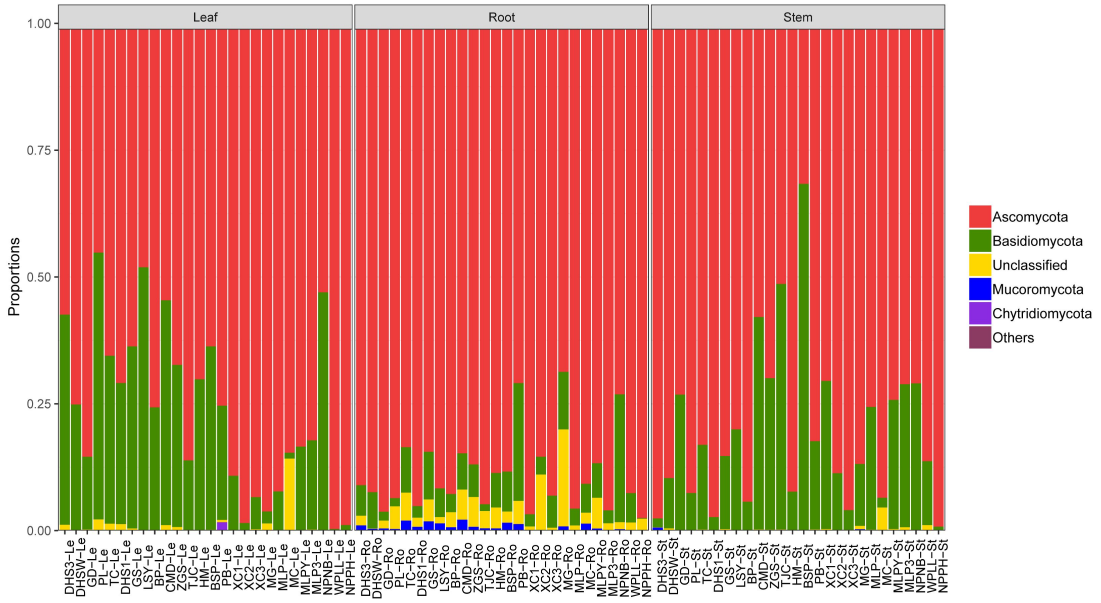

Fig. S12 The relative abundance of fungal taxa at the phylum level within plant compartments in *P. notoginseng*. BP, Banpo village; BSP, Beishapo village; CMD, Chengmendong village; DHS1, Daheishan village 1; DHS3, Daheishan village 3; DHSW, Daheishan village (no pesticide); GD, Gende village; GS, Guishan village; HM, Hemo village; LSY, Laosaiyi village; MC, Meichang village; MG, Maguan village; MLP1, Masupo village 1; MLP3, Masupo village 3; MLPY, Masupo village; NPNB, Naponabing village; NPPH, Napopohe village; PB, Pingba village; PL, Panlong village; TC, Tiechang village; TJC, Tongjiachong village; WPLL, Wupingleilong village; XC1, Xichou village; XC2, Xichou village 2; XC3, Xichou village 3; ZGS, Zhiguoshan village.

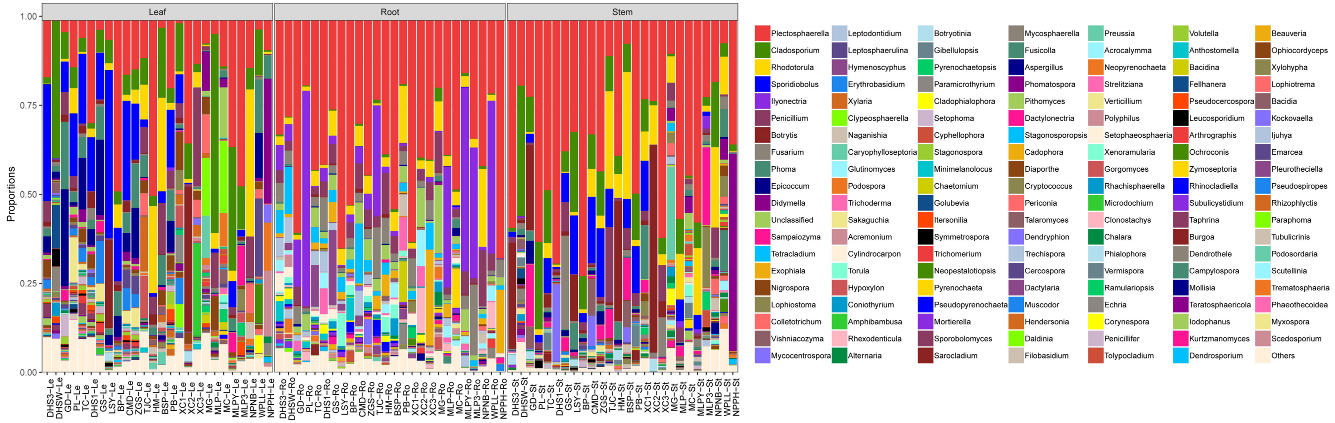

Fig. S13 The relative abundance of fungal taxa at the genus level within plant compartments in *P. notoginseng*. BP, Banpo village; BSP, Beishapo village; CMD, Chengmendong village; DHS1, Daheishan village 1; DHS3, Daheishan village 3; DHSW, Daheishan village (no pesticide); GD, Gende village; GS, Guishan village; HM, Hemo village; LSY, Laosaiyi village; MC, Meichang village; MG, Maguan village; MLP1, Masupo village 1; MLP3, Masupo village 3; MLPY, Masupo village; NPNB, Naponabing village; NPPH, Napopohe village; PB, Pingba village; PL, Panlong village; TC, Tiechang village; TJC, Tongjiachong village; WPLL, Wupingleilong village; XC1, Xichou village; XC2, Xichou village 2; XC3, Xichou village 3; ZGS, Zhiguoshan village.

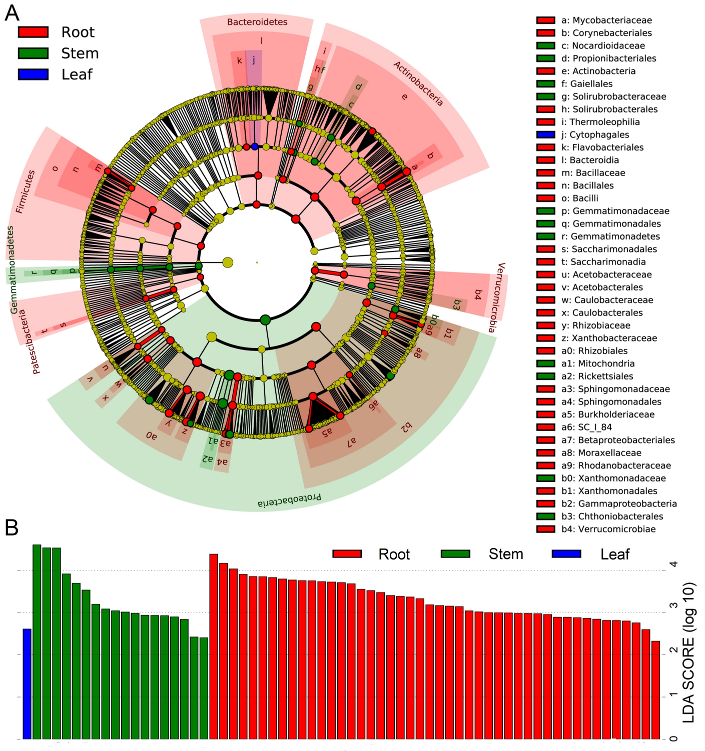

Fig. S14 Linear discriminant effect size-identified differentially abundant bacterial taxa within plant compartments in *P. notoginseng*. (A). Cladograms are shown for the LDA values exceeding 2.0 for bacterial clarity. Small circles and shading with different colors in the diagram represent the abundance of taxa in the different plant samples. Yellow circles present non-significant differences in abundance between the samples of a particular taxon. Each circle's diameter is proportional to the taxon abundance. (B). Enriched taxa reaching a LDA significance threshold of 2.

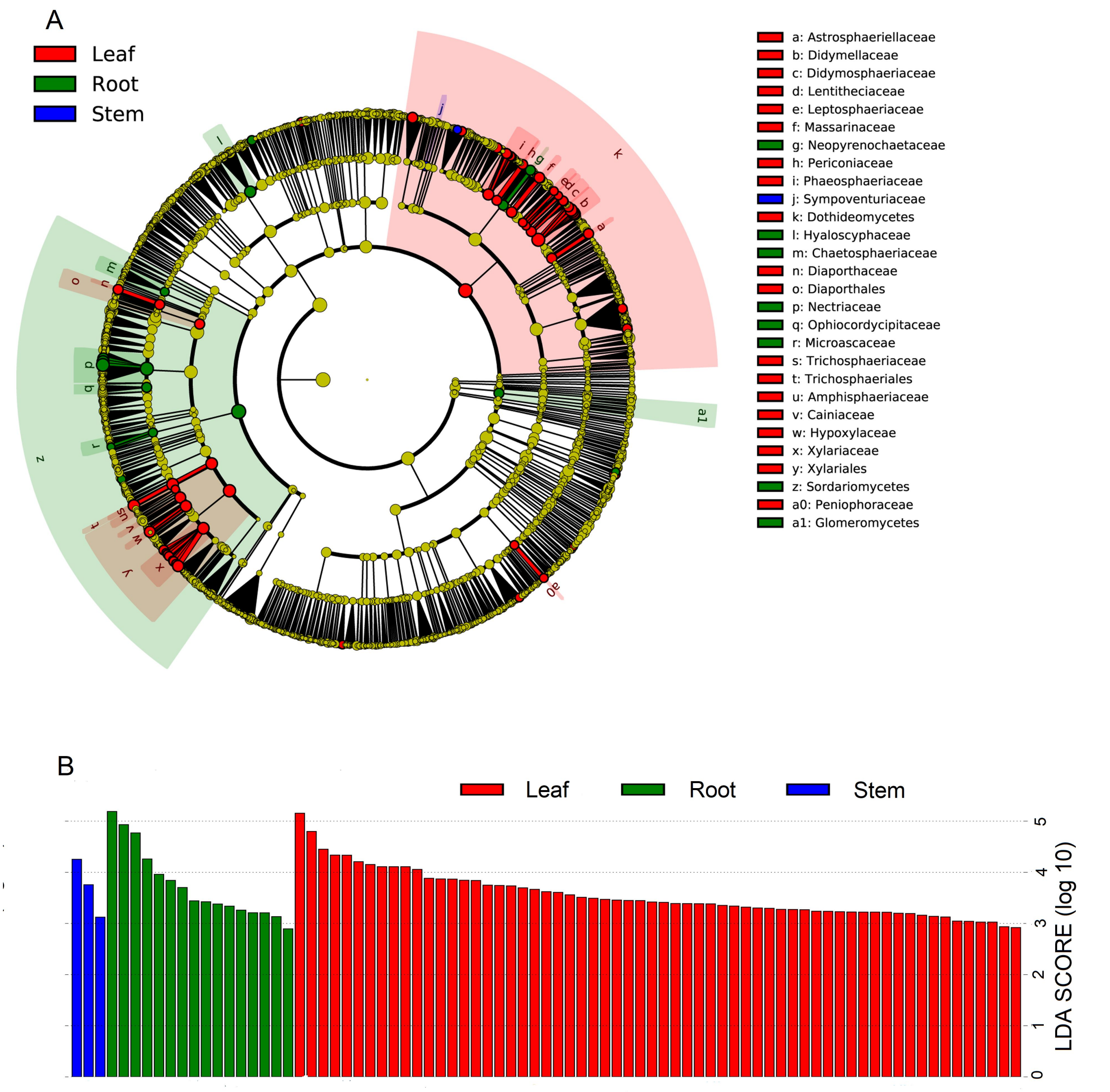

Fig. S15 Linear discriminant effect size-identified differentially abundant fungal taxa within plant compartments in *P. notoginseng*. (A). Cladograms are shown for the LDA values exceeding 3.0 for bacterial clarity. Small circles and shading with different colors in the diagram represent the abundance of taxa in the different plant samples. Yellow circles present non-significant differences in abundance between the samples of a particular taxon. Each circle's diameter is proportional to the taxon abundance. (B). Enriched taxa reaching a LDA significance threshold of 3.

Table S1 Bacterial barcodes and sequences numbers within plant compartments in *P. notoginseng*. BP: Banpo village; BSP: Beishapo village; CMD: Chengmendong village; DHS1: Daheishan village 1; DHS3: Daheishan village 3; DHSW: Daheishan village (no pesticide); GD: Gende village; GS: Guishan village; HM: Hemo village; LSY: Laosaiyi village; MC: Meichang village; MG: Maguan village; MLP1: Masupo village 1; MLP3: Masupo village 3; MLPY: Masupo village; NPNB: Naponabing village; NPPH: Napopohe village; PB: Pingba village; PL: Panlong village; TC: Tiechang village; TJC: Tongjiachong village; WPLL: Wupingleilong village; XC1: Xichou village; XC2: Xichou village 2; XC3: Xichou village 3; ZGS: Zhiguoshan village.

Samples	Barcodes	Sequences numbers	Samples	Barcodes	Sequences numbers	Samples	Barcodes	Sequences numbers
BP-Ro-1	AGGCTCTA	25308	BP-St-1	AGGCTCTA	26451	BP-Le-1	AGGCTCTA	25124
BP-Ro-2	AAGCTCAT	33368	BP-St-2	AAGCTCAT	52622	BP-Le-2	AAGCTCAT	29047
BP-Ro-3	ACAGCCAG	19706	BP-St-3	ACAGCCAG	19572	BP-Le-3	ACAGCCAG	11559
BSP-Ro-1	ACGAGTGA	15620	BSP-St-1	ACGAGTGA	15513	BSP-Le-1	ACGAGTGA	14270
BSP-Ro-2	ACTGACGA	16833	BSP-St-2	ACTGACGA	13752	BSP-Le-2	ACTGACGA	14988
BSP-Ro-3	AGAGTTGA	22806	BSP-St-3	AGAGTTGA	20018	BSP-Le-3	AGAGTTGA	19268
CMD-Ro-1	ACCTGCAT	26472	CMD-St-1	ACCTGCAT	19140	CMD-Le-1	ACCTGCAT	33277
CMD-Ro-2	ACGTCCAT	13473	CMD-St-2	ACGTCCAT	11795	CMD-Le-2	ACGTCCAT	21965
CMD-Ro-3	AGAGCATG	23966	CMD-St-3	AGAGCATG	26881	CMD-Le-3	AGAGCATG	20775
DHS1-Ro-1	AGACTAGT	44469	DHS1-St-1	AGACTAGT	44595	DHS1-Le-1	AGACTAGT	58351
DHS1-Ro-2	AGCAGATG	21397	DHS1-St-2	AGCAGATG	16301	DHS1-Le-2	AGCAGATG	29393
DHS1-Ro-3	AGGACCAT	31169	DHS1-St-3	AGGACCAT	53789	DHS1-Le-3	AGGACCAT	28512
DHS3-Ro-1	AACGCACG	37435	DHS3-St-1	AACGCACG	27737	DHS3-Le-1	AACGCACG	20140
DHS3-Ro-2	ACACTGCT	47210	DHS3-St-2	ACACTGCT	29153	DHS3-Le-2	ACACTGCT	23861
DHS3-Ro-3	ACCACCCT	37315	DHS3-St-3	ACCACCCT	20876	DHS3-Le-3	ACCACCCT	21819

DHSW-Ro-1	ACGCTCGT	27327	DHSW-St-1	ACGCTCGT	22537	DHSW-Le-1	ACGCTCGT	19517
DHSW-Ro-2	ACTCATAC	29756	DHSW-St-2	ACTCATAC	28990	DHSW-Le-2	ACTCATAC	19468
DHSW-Ro-3	AGACCCTG	14520	DHSW-St-3	AGACCCTG	10337	DHSW-Le-3	AGACCCTG	8315
GD-Ro-1	AGCACGCT	38322	GD-St-1	AGCACGCT	33764	GD-Le-1	AGCACGCT	25355
GD-Ro-2	ACAGAGGT	32528	GD-St-2	ACAGAGGT	30898	GD-Le-2	ACAGAGGT	41919
GD-Ro-3	ACCAGCTT	26045	GD-St-3	ACCAGCTT	27528	GD-Le-3	ACCAGCTT	32223
GS-Ro-1	AAGAGGCT	17504	GS-St-1	AAGAGGCT	28361	GS-Le-1	AAGAGGCT	23247
GS-Ro-2	ACCACCGA	23648	GS-St-2	ACCACCGA	37567	GS-Le-2	ACCACCGA	27094
GS-Ro-3	ACGTAGGA	17479	GS-St-3	ACGTAGGA	18092	GS-Le-3	ACGTAGGA	8705
HM-Ro-1	ATGCTACA	21300	HM-St-1	ATGCTACA	27228	HM-Le-1	ATGCTACA	15409
HM-Ro-2	ATCATCTA	23444	HM-St-2	ATCATCTA	16561	HM-Le-2	ATCATCTA	18459
HM-Ro-3	AATCGACA	27928	HM-St-3	AATCGACA	43541	HM-Le-3	AATCGACA	28800
LSY-Ro-1	ACTCGCGA	34990	LSY-St-1	ACTCGCGA	39052	LSY-Le-1	ACTCGCGA	14316
LSY-Ro-2	ACGAATCC	26506	LSY-St-2	ACGAATCC	36425	LSY-Le-2	ACGAATCC	26807
LSY-Ro-3	AGCATACT	60776	LSY-St-3	AGCATACT	27430	LSY-Le-3	AGCATACT	27742
MC-Ro-1	AGATCGCT	25806	MC-St-1	AGATCGCT	73136	MC-Le-1	AGATCGCT	51612
MC-Ro-2	AGCTATCA	25071	MC-St-2	AGCTATCA	27502	MC-Le-2	AGCTATCA	19280
MC-Ro-3	AGTCCTTA	28846	MC-St-3	AGTCCTTA	36427	MC-Le-3	AGTCCTTA	35500
MG-Ro-1	AGCGTTCA	34272	MG-St-1	AGCGTTCA	32628	MG-Le-1	AGCGTTCA	26907
MG-Ro-2	ATTTGTCA	28883	MG-St-2	ATTTGTCA	31422	MG-Le-2	ATTTGTCA	28755
MG-Ro-3	ACATGGTA	18120	MG-St-3	ACATGGTA	22706	MG-Le-3	ACATGGTA	23348
MLP-Ro-1	ACGCGTAC	17691	MLP-St-1	ACGCGTAC	15175	MLP-Le-1	ACGCGTAC	18482
MLP-Ro-2	ACTACTGA	20446	MLP-St-2	ACTACTGA	27890	MLP-Le-2	ACTACTGA	21393
MLP-Ro-3	ACTGTCTA	22902	MLP-St-3	ACTGTCTA	20537	MLP-Le-3	ACTGTCTA	19323
MLP3-Ro-1	AGATGCTC	38246	MLP3-St-1	AGATGCTC	42160	MLP3-Le-1	AGATGCTC	38213
MLP3-Ro-2	AGCTCGTA	20458	MLP3-St-2	AGCTCGTA	38965	MLP3-Le-2	AGCTCGTA	27218

MLP3-Ro-3	ACACCTAG	24782	MLP3-St-3	ACACCTAG	46075	MLP3-Le-3	ACACCTAG	37206
MLPY-Ro-1	ACACGTCA	14614	MLPY-St-1	ACACGTCA	29577	MLPY-Le-1	ACACGTCA	21003
MLPY-Ro-2	ACATTCGT	30933	MLPY-St-2	ACATTCGT	31261	MLPY-Le-2	ACATTCGT	15248
MLPY-Ro-3	ACTTGCGA	29373	MLPY-St-3	ACTTGCGA	20253	MLPY-Le-3	ACTTGCGA	31746
NPNB-Ro-1	ACTATCGA	19016	NPNB-St-1	ACTATCGA	32126	NPNB-Le-1	ACTATCGA	11434
NPNB-Ro-2	AGAACCGC	25205	NPNB-St-2	AGAACCGC	38356	NPNB-Le-2	AGAACCGC	17660
NPNB-Ro-3	AGTGACTC	26554	NPNB-St-3	AGTGACTC	29949	NPNB-Le-3	AGTGACTC	15579
NPPH-Ro-1	ATCCTGCC	37103	NPPH-St-1	ATCCTGCC	29945	NPPH-Le-1	ATCCTGCC	17527
NPPH-Ro-2	AGTGCTAC	22581	NPPH-St-2	AGTGCTAC	17353	NPPH-Le-2	AGTGCTAC	22895
NPPH-Ro-3	ATACTCGT	31090	NPPH-St-3	ATACTCGT	44660	NPPH-Le-3	ATACTCGT	23046
PB-Ro-1	AGCGATCA	20593	PB-St-1	AGCGATCA	40530	PB-Le-1	AGCGATCA	13421
PB-Ro-2	AGTACGTA	25259	PB-St-2	AGTACGTA	33899	PB-Le-2	AGTACGTA	22142
PB-Ro-3	ACAATTCA	16219	PB-St-3	ACAATTCA	27681	PB-Le-3	ACAATTCA	17116
PL-Ro-1	ACGGAGCT	24208	PL-St-1	ACGGAGCT	18607	PL-Le-1	ACGGAGCT	13630
PL-Ro-2	AGCTTCGT	44876	PL-St-2	AGCTTCGT	17786	PL-Le-2	AGCTTCGT	22552
PL-Ro-3	AACTGCTA	38801	PL-St-3	AACTGCTA	29474	PL-Le-3	AACTGCTA	22102
TC-Ro-1	ACCGCGCT	27779	TC-St-1	ACCGCGCT	24719	TC-Le-1	ACCGCGCT	32715
TC-Ro-2	ACGGTCTG	10118	TC-St-2	ACGGTCTG	23843	TC-Le-2	ACGGTCTG	17145
TC-Ro-3	ACAGGACT	41988	TC-St-3	ACAGGACT	34878	TC-Le-3	ACAGGACT	27697
TJC-Ro-1	ACGACTCT	18049	TJC-St-1	ACGACTCT	18824	TJC-Le-1	ACGACTCT	38339
TJC-Ro-2	ACGTGCTA	8268	TJC-St-2	ACGTGCTA	9296	TJC-Le-2	ACGTGCTA	13935
TJC-Ro-3	ATACGCGA	38019	TJC-St-3	ATACGCGA	21760	TJC-Le-3	ATACGCGA	31816
WPLL-Ro-1	ATGGTCTA	26789	WPLL-St-1	ATGGTCTA	43293	WPLL-Le-1	ATGGTCTA	19989
WPLL-Ro-2	AGGTTGCT	22188	WPLL-St-2	AGGTTGCT	34807	WPLL-Le-2	AGGTTGCT	21602
WPLL-Ro-3	CCAGTAAT	13481	WPLL-St-3	CCAGTAAT	20685	WPLL-Le-3	CCAGTAAT	8958
XC1-Ro-1	ACGATCGA	15159	XC1-St-1	ACGATCGA	17273	XC1-Le-1	ACGATCGA	12223

XC1-Ro-2	ACGTTGCT	8571	XC1-St-2	ACGTTGCT	17351	XC1-Le-2	ACGTTGCT	8358
XC1-Ro-3	ATAGTCGT	24563	XC1-St-3	ATAGTCGT	34686	XC1-Le-3	ATAGTCGT	13158
XC2-Ro-1	AGATACTA	32572	XC2-St-1	AGATACTA	33940	XC2-Le-1	AGATACTA	29493
XC2-Ro-2	AGCGCACT	45060	XC2-St-2	AGCGCACT	37555	XC2-Le-2	AGCGCACT	41693
XC2-Ro-3	ACACAGTA	25652	XC2-St-3	ACACAGTA	31840	XC2-Le-3	ACACAGTA	25633
XC3-Ro-1	AGTTGCTC	34726	XC3-St-1	AGTTGCTC	31241	XC3-Le-1	AGTTGCTC	26069
XC3-Ro-2	ACGCATCA	16202	XC3-St-2	ACGCATCA	19024	XC3-Le-2	ACGCATCA	21080
XC3-Ro-3	AAGCCCTG	22191	XC3-St-3	AAGCCCTG	24574	XC3-Le-3	AAGCCCTG	21609
ZGS-Ro-1	AGCCAACT	33391	ZGS-St-1	AGCCAACT	26196	ZGS-Le-1	AGCCAACT	36043
ZGS-Ro-2	AGGTGTAC	27681	ZGS-St-2	AGGTGTAC	22096	ZGS-Le-2	AGGTGTAC	22704
ZGS-Ro-3	AATCATCG	28410	ZGS-St-3	AATCATCG	34202	ZGS-Le-3	AATCATCG	20888

Ro, St, Le represent root, stem, leaf, respectively; -1,-2 and -3 represent three replicates in one site.

Table S2 Fungal barcodes and sequences numbers within plant compartments in *P. notoginseng*. BP: Banpo village; BSP: Beishapo village; CMD: Chengmendong village; DHS1: Daheishan village 1; DHS3: Daheishan village 3; DHSW: Daheishan village (no pesticide); GD: Gende village; GS: Guishan village; HM: Hemo village; LSY: Laosaiyi village; MC: Meichang village; MG: Maguan village; MLP1: Masupo village 1; MLP3: Masupo village 3; MLPY: Masupo village; NPNB: Naponabing village; NPPH: Napopohe village; PB: Pingba village; PL: Panlong village; TC: Tiechang village; TJC: Tongjiachong village; WPLL: Wupingleilong village; XC1: Xichou village; XC2: Xichou village 2; XC3: Xichou village 3; ZGS: Zhiguoshan village.

Samples	F-Barcodes	R-Barcodes	Sequences numbers	Samples	F-Barcodes	R-Barcodes	Sequences numbers	Samples	F-Barcodes	R-Barcodes	Sequences numbers
BP-Ro-1	CATCGT	TTCAGA	61131	BP-St-1	CTACGTA	GTCTG	55849	BP-Le-1	AACCGAGA	TCTCAGTC	74651
BP-Ro-2	TAGCGGA	CATAAGT	68981	BP-St-2	GAGCA	CGATGCA	74047	BP-Le-2	TCTGTGA	CGCTGAT	53270
BP-Ro-3	ACGTGTT	GTCGATT	54654	BP-St-3	TGACCA	ACGATT	68372	BP-Le-3	AACAT	TACTATG	54296
BSP-Ro-1	TAGCATGC	CCATGGGT	52532	BSP-St-1	AGCACAT	TATAG	68090	BSP-Le-1	CTTGTA	GCCAAT	70371
BSP-Ro-2	CTGTA	TACAT	63762	BSP-St-2	CATTC	GTACATT	58275	BSP-Le-2	GCGAAGT	CGAGG	55076
BSP-Ro-3	GAGATA	AGTGGA	61270	BSP-St-3	GTAGAG	CGTACG	74597	BSP-Le-3	TCATT	ATGAGTC	62568
CMD-Ro-1	ACGACTAC	GGATTGGT	56212	CMD-St-1	TCACGTC	ACTCG	54375	CMD-Le-1	AGTTCC	TGTCGT	59042
CMD-Ro-2	TAGCGGA	CATAAGT	57379	CMD-St-2	AGTCA	TCGTAGA	54698	CMD-Le-2	CAGAGAT	GCTGG	59418
CMD-Ro-	CTAGC	TCACC	68885	CMD-St-3	ACCGT	GTCAA	62888	CMD-Le-3	GCGTT	CATGATA	59522

3											
DHS1-Ro-1	GTACTT	ATGCCT	67137	DHS1-St-1	TGGCTA	CCAGCT	67006	DHS1-Le-1	ATGTGCT	TAAGA	57217
DHS1-Ro-2	AGGAT	CGCTT	54766	DHS1-St-2	CTACGGA	TCGAAGA	54618	DHS1-Le-2	CAATT	GCTGTGA	73637
DHS1-Ro-3	TAACGA	GCCAGT	62251	DHS1-St-3	GCTGTGGA	ACGTGGTA	71357	DHS1-Le-3	GCAGGA	CGGAAT	51301
DHS3-Ro-1	AACCGAGA	TCTCAGTC	54594	DHS3-St-1	ATCGTA	GCTCTA	68452	DHS3-Le-1	TGTCTAC	ATACG	55402
DHS3-Ro-2	TCTGTGA	CGCTGAT	56321	DHS3-St-2	TGCGA	CATCT	64135	DHS3-Le-2	ACGCA	TATCTGA	57492
DHS3-Ro-3	AACAT	TACTATG	70073	DHS3-St-3	CGCCTTAT	TGGTACGT	64600	DHS3-Le-3	CGATGT	GTGAAA	72410
DHSW-Ro-1	CTTGTA	GCCAAT	65180	DHSW-St-1	GAATTCA	AACGCCT	57540	DHSW-Le-1	GACGCTG	CGCAC	74954
DHSW-Ro-2	GCGAAGT	CGAGG	56206	DHSW-St-2	ATGAAAC	GAACTTC	62218	DHSW-Le-2	TATCC	ACCTTCT	70416
DHSW-Ro-3	TCATT	ATGAGTC	53925	DHSW-St-3	TATCGGGA	CCGGATAT	74688	DHSW-Le-3	ACAGTG	TATAAT	72658
GD-Ro-1	AGTTCC	TGTCGT	73555	GD-St-1	CATCGT	TTCAGA	69335	GD-Le-1	CTACGTA	GTCTG	53446
GD-Ro-2	CAGAGAT	GCTGG	51517	GD-St-2	GCTTA	ATTGA	68526	GD-Le-2	GAGCA	CGATGCA	65457
GD-Ro-3	GCGTT	CATGATA	71248	GD-St-3	ACGTGTT	GTCGATT	51943	GD-Le-3	TGACCA	ACGATT	69567
GS-Ro-1	ATGTGCT	TAAGA	71599	GS-St-1	TAGCATGC	CCATGGGT	55834	GS-Le-1	AGCACAT	TATAG	74028

GS-Ro-2	CAATT	GCTGTGA	67666	GS-St-2	CTGTA	TACAT	61905	GS-Le-2	CATTC	GTACATT	62316
GS-Ro-3	GCAGGA	CGGAAT	55525	GS-St-3	GAGATA	AGTGGA	61332	GS-Le-3	GTAGAG	CGTACG	62744
HM-Ro-1	TGTCTAC	ATACG	53278	HM-St-1	ACGACTAC	GGATTGGT	59165	HM-Le-1	TCACGTC	ACTCG	55367
HM-Ro-2	ACGCA	TATCTGA	55062	HM-St-2	TAGCGGA	CATAAGT	62699	HM-Le-2	AGTCA	TCGTAGA	56028
HM-Ro-3	CGATGT	GTGAAA	54723	HM-St-3	CTAGC	TCACC	71952	HM-Le-3	ACCGT	GTCAA	50437
LSY-Ro-1	GACGCTG	CGCAC	62082	LSY-St-1	GTACTT	ATGCCT	53555	LSY-Le-1	TGGCTA	CCAGCT	64270
LSY-Ro-2	TATCC	ACCTTCT	67759	LSY-St-2	AGGAT	CGCTT	63545	LSY-Le-2	CTACGGA	TCGAAGA	74474
LSY-Ro-3	ACAGTG	TATAAT	72739	LSY-St-3	TAACGA	GCCAGT	67874	LSY-Le-3	GCTGTGGA	ACGTGGTA	50332
MC-Ro-1	CTACGTA	GTCTG	51132	MC-St-1	AACCGAGA	TCTCAGTC	73015	MC-Le-1	ATCGTA	GCTCTA	54706
MC-Ro-2	GAGCA	CGATGCA	68103	MC-St-2	TCTGTGA	CGCTGAT	62789	MC-Le-2	TGCGA	CATCT	72471
MC-Ro-3	TGACCA	ACGATT	67457	MC-St-3	AACAT	TACTATG	52273	MC-Le-3	CGCCTTAT	TGGTACGT	66249
MG-Ro-1	AGCACAT	TATAG	62472	MG-St-1	CTTGTA	GCCAAT	74330	MG-Le-1	GAATTCA	AACGCCT	61967
MG-Ro-2	CATTC	GTACATT	65919	MG-St-2	GCGAAGT	CGAGG	50672	MG-Le-2	ATGAAAC	GAACTTC	73139
MG-Ro-3	GTAGAG	CGTACG	62706	MG-St-3	TCATT	ATGAGTC	59489	MG-Le-3	TATCGGGA	CCGGATAT	59904
MLP-Ro-1	TCACGTC	ACTCG	74994	MLP-St-1	AGTTCC	TGTCGT	57432	MLP-Le-1	CATCGT	TTCAGA	64868
MLP-Ro-2	AGTCA	TCGTAGA	71809	MLP-St-2	CAGAGAT	GCTGG	57683	MLP-Le-2	AGGAT	CGCTT	68268
MLP-Ro-3	ACCGT	GTCAA	51008	MLP-St-3	GCGTT	CATGATA	56881	MLP-Le-3	ACGTGT	GTCGATT	66477

									T		
MLP3-Ro-1	TGGCTA	CCAGCT	68894	MLP3-St-1	ATGTGCT	TAAGA	71503	MLP3-Le-1	TAGCATGC	CCATGGGT	62807
MLP3-Ro-2	CTACGGA	TCGAAGA	50806	MLP3-St-2	CAATT	GCTGTGA	68394	MLP3-Le-2	CTGTA	TACAT	57765
MLP3-Ro-3	GCTGTGGA	ACGTGGTA	65683	MLP3-St-3	GCAGGA	CGGAAT	52177	MLP3-Le-3	AACCGAGA	TCTCAGTC	54066
MLPY-Ro-1	ATCGTA	GCTCTA	63419	MLPY-St-1	TGTCTAC	ATACG	59355	MLPY-Le-1	ACGACTAC	GGATTGGT	63202
MLPY-Ro-2	TGCGA	CATCT	50391	MLPY-St-2	ACGCA	TATCTGA	73992	MLPY-Le-2	TAGCGGA	CATAAGT	61653
MLPY-Ro-3	CGCCTTAT	TGGTACGT	52213	MLPY-St-3	CGATGT	GTGAAA	66501	MLPY-Le-3	CTAGC	TCACC	59771
NPNB-Ro-1	GAATTCA	AACGCCT	67656	NPNB-St-1	GACGCTG	CGCAC	56332	NPNB-Le-1	GTACTT	ATGCCT	69989
NPNB-Ro-2	ATGAAAC	GAACTTC	71342	NPNB-St-2	TATCC	ACCTTCT	70207	NPNB-Le-2	AGGAT	CGCTT	54560
NPNB-Ro-3	TATCGGGA	CCGGATAT	69048	NPNB-St-3	ACAGTG	TATAAT	65007	NPNB-Le-3	TAACGA	GCCAGT	65932
NPPH-Ro-1	CATCGT	TTCAGA	72876	NPPH-St-1	CTACGTA	GTCTG	64863	NPPH-Le-1	AACCGAGA	TCTCAGTC	61986
NPPH-Ro-2	CTAGC	TCACC	52748	NPPH-St-2	GAGCA	CGATGCA	70454	NPPH-Le-2	TCTGTGA	CGCTGAT	72755
NPPH-Ro-3	ACGTGTT	GTCGATT	57249	NPPH-St-3	TGACCA	ACGATT	72874	NPPH-Le-3	AACAT	TACTATG	51721
PB-Ro-1	TAGCAT	CCATGGG	52629	PB-St-1	AGCACAT	TATAG	51087	PB-Le-1	CTTGTA	GCCAAT	62110

	GC	T									
PB-Ro-2	CTGTA	TACAT	67839	PB-St-2	CATTC	GTACATT	72157	PB-Le-2	GCGAAGT	CGAGG	50699
PB-Ro-3	GAGATA	AGTGGA	66641	PB-St-3	GTAGAG	CGTACG	57382	PB-Le-3	TCATT	ATGAGTC	67717
PL-Ro-1	ACGACTAC	GGATTGGT	67187	PL-St-1	TCACGTC	ACTCG	54175	PL-Le-1	AGTTCC	TGTCGT	68448
PL-Ro-2	TAGCGGA	CATAAGT	60724	PL-St-2	AGTCA	TCGTAGA	71228	PL-Le-2	CAGAGAT	GCTGG	64917
PL-Ro-3	CTAGC	TCACC	55066	PL-St-3	ACCGT	GTCAA	52562	PL-Le-3	GCGTT	CATGATA	52526
TC-Ro-1	GTACTT	ATGCCT	57779	TC-St-1	TGGCTA	CCAGCT	61957	TC-Le-1	ATGTGCT	TAAGA	63814
TC-Ro-2	AGGAT	CGCTT	63472	TC-St-2	CTACGGA	TCGAAGA	69615	TC-Le-2	CAATT	GCTGTGA	69365
TC-Ro-3	TAACGA	GCCAGT	68297	TC-St-3	GCTGTGGA	ACGTGGTA	61401	TC-Le-3	GCAGGA	CGGAAT	64429
TJC-Ro-1	AACCGAGA	TCTCAGTC	63488	TJC-St-1	ATCGTA	GCTCTA	69713	TJC-Le-1	TGTCTAC	ATACG	64077
TJC-Ro-2	TCTGTGA	CGCTGAT	65706	TJC-St-2	TGCGA	CATCT	62376	TJC-Le-2	ACGCA	TATCTGA	53104
TJC-Ro-3	AACAT	TACTATG	60840	TJC-St-3	CGCCTTAT	TGGTACGT	55292	TJC-Le-3	CGATGT	GTGAAA	54709
WPLL-Ro-1	CTTGTA	GCCAAT	71679	WPLL-St-1	GAATTCA	AACGCCT	65500	WPLL-Le-1	GACGCTG	CGCAC	67870
WPLL-Ro-2	GCGAAGT	CGAGG	71641	WPLL-St-2	ATGAAAC	GAACTTC	66487	WPLL-Le-2	TATCC	ACCTTCT	57080
WPLL-Ro-3	TCATT	ATGAGTC	55216	WPLL-St-3	TATCGGGA	CCGGATAT	55606	WPLL-Le-3	ACAGTG	TATAAT	50714
XC1-Ro-1	AGTTCC	TGTCGT	52713	XC1-St-1	CATCGT	TTCAGA	54244	XC1-Le-1	CTACGT	GTCTG	59253

									A		
XC1-Ro-2	CAGAGAT	GCTGG	74910	XC1-St-2	GCTTA	ATTGA	70836	XC1-Le-2	GAGCA	CGATGCA	73691
XC1-Ro-3	GCGTT	CATGATA	53851	XC1-St-3	ACGTGTT	GTCGATT	69703	XC1-Le-3	TGACCA	ACGATT	58735
XC2-Ro-1	ATGTGCT	TAAGA	71483	XC2-St-1	TAGCATGC	CCATGGGT	67161	XC2-Le-1	AGCACAT	TATAG	62949
XC2-Ro-2	CAATT	GCTGTGA	61469	XC2-St-2	CTGTA	TACAT	71549	XC2-Le-2	CATTC	GTACATT	57846
XC2-Ro-3	GCAGGA	CGGAAT	57570	XC2-St-3	GAGATA	AGTGGA	67628	XC2-Le-3	GTAGAG	CGTACG	69760
XC3-Ro-1	TGTCTAC	ATACG	54691	XC3-St-1	ACGACTAC	GGATTGGT	60617	XC3-Le-1	TCACGTC	ACTCG	69601
XC3-Ro-2	ACGCA	TATCTGA	53224	XC3-St-2	TAGCGGA	CATAAGT	67951	XC3-Le-2	AGTCA	TCGTAGA	57657
XC3-Ro-3	CGATGT	GTGAAA	67993	XC3-St-3	CTAGC	TCACC	62287	XC3-Le-3	ACCGT	GTCAA	67964
ZGS-Ro-1	GACGCTG	CGCAC	69535	ZGS-St-1	GTACTT	ATGCCT	56464	ZGS-Le-1	TGGCTA	CCAGCT	62661
ZGS-Ro-2	TATCC	ACCTTCT	51752	ZGS-St-2	AGGAT	CGCTT	63915	ZGS-Le-2	CTACGGA	TCGAAGA	54661
ZGS-Ro-3	ACAGTG	TATAAT	55149	ZGS-St-3	TAACGA	GCCAGT	62922	ZGS-Le-3	GCTGTGGA	ACGTGGTA	56581

Ro, St, Le represent root, stem, leaf, respectively; -1,-2 and -3 represent three replicates in one site.

Table S3 Alpha diversity of bacterial communities within plant compartments in *P. notoginseng* (x±SD).

Alpha diversity	Root	*P*	Stem	*P*	Leaf	*P*	F (2,233)	*P*-value
Chao 1	1248.30±388.85	a	737.38±536.64	b	552.95±467.18	c	46.19	0.0001
OTU	522.04±173.73	a	317.46±251.51	b	207.97±200.96	c	44.44	0.0001
Shannon	3.07±0.70	a	2.53±0.92	b	1.96±0.78	c	35.52	0.0001

Table S4 Phyla composition of bacterial communities within plant compartments in *P. notoginseng* (x±SD).

Phyla	Root (%)	*P*	Stem (%)	*P*	Leaf (%)	*P*	F (2,233)	*P*-value
Cyanobacteria	49.44±6.91	b	47.73±7.53	b	57.97±7.22	a	44.42	0.000
Proteobacteria	45.88±5.97	b	47.86±6.07	a	39.78±6.42	c	36.08	0.000
Actinobacteria	2.16±1.82	a	2.07±3.54	a	0.88±0.32	b	4.552	0.012
Unclassified	0.18±0.002	b	0.67±1.91	a	0.21±0.39	b	4.56	0.011
Acidobacteria	0.40±0.37	a	0.32±0.74	a	0.25±0.66	a	1.23	0.295
Bacteroidetes	0.36±0.24	a	0.34±0.49	a	0.24±0.59	a	1.43	0.241
Chloroflexi	0.26±0.23	a	0.24±0.49	a	0.21±0.42	a	0.35	0.708
Verrucomicrobia	0.33±0.38	a	0.23±0.47	b	0.10±0.16	c	7.91	0.000
Planctomycetes	0.43±0.31	a	0.09±0.17	b	0.09±0.19	b	54.29	0.000
Gemmatimonadetes	0.11±0.12	ab	0.21±0.50	a	0.09±0.19	b	2.97	0.053
Firmicutes	0.19±0.35	a	0.08±0.13	b	0.04±0.08	b	9.71	0.000
Deinococcus-Thermus	0.00±0.00	a	0.01±0.03	a	0.04±0.24	a	2.56	0.079
Others	0.26±0.20	a	0.15±0.29	b	0.10±0.25	b	8.29	0.000

Table S5 Alpha diversity of fungal communities within plant compartments in *P. notoginseng* (x±SD).

Alpha diversity	Root	*P*	Stem	*P*	Leaf	*P*	F (2,233)	*P*-value
Chao 1	517.48±121.90	b	596.48±234.76	b	981.08±524.81	a	41.67	0.0001
OTU	436.32±111.38	b	489.13±202.93	b	803.45±452.44	a	35.69	0.0001
Shannon	2.83±0.62	b	2.64±0.83	b	3.23±0.89	a	11.32	0.0001

Table S6 Phyla composition of fungal communities within plant compartments in *P. notoginseng* (x±SD).

Phyla	Root (%)	*P*	Stem (%)	*P*	Leaf (%)	*P*	F (2,233)	*P*-value
Ascomycota	88.92±11.07	a	80.41±19.60	b	75.94±18.41	b	12.04	0.000
Basidiomycota	6.62±1.73	b	19.16±2.23	a	23.00±18.61	a	21.85	0.000
Chytridiomycota	0.00±0.00	a	0.00±0.00	a	0.06±0.00	a	0.92	0.398
Cryptomycota	0±0	a	0.00±0.00	a	0.00±0.00	a	1.70	0.184
Mucoromycota	0.72±0.10	a	0.05±0.01	b	0.01±0.00	b	48.80	0.000
Olpidiomycota	0.00±0.00	a	0±0	b	0±0	b	3.99	0.020
Unclassified	3.74±1.73	a	0.38±0.10	b	0.99±0.20	b	12.31	0.000
Zoopagomycota	0.00±0.00	a	0.00±0.00	a	0.00±0.00	a	5.24	0.006

Table S7 Topological properties of co-occurring bacterial networks within plant compartments calculated using the statistical Cytoscape package.

Network properties	Root	Stem	Leaf
Number of nodes	246	380	388
Number of edges	779	1659	1902
Positive edges	779	1659	1902
Negative edges	0	0	0
Modularity	0.80	0.72	0.75
Number of communities	23	30	31
Network diameter	21	15	19
Average path length	7.39	4.82	5.74
Average degree	6.33	8.73	9.80
Average clustering coefficient	0.64	0.60	0.66

Table S8 Topological properties of co-occurring fungal networks within plant compartments calculated using the statistical Cytoscape package.

Network properties	Root	Stem	Leaf
Number of nodes	221	273	416
Number of edges	431	685	1175
Positive edges	431	685	1175
Negative edges	0	0	0
Modularity	0.95	0.92	0.91
Number of communities	58	63	54
Network diameter	2	4	11
Average path length	1.02	1.60	3.99
Average degree	1.95	2.51	2.83
Average clustering coefficient	0.99	0.93	0.72
